# Enzymatic Removal of Ribonucleotides from DNA Is Essential for Mammalian Genome Integrity and Development

**DOI:** 10.1016/j.cell.2012.04.011

**Published:** 2012-05-25

**Authors:** Martin A.M. Reijns, Björn Rabe, Rachel E. Rigby, Pleasantine Mill, Katy R. Astell, Laura A. Lettice, Shelagh Boyle, Andrea Leitch, Margaret Keighren, Fiona Kilanowski, Paul S. Devenney, David Sexton, Graeme Grimes, Ian J. Holt, Robert E. Hill, Martin S. Taylor, Kirstie A. Lawson, Julia R. Dorin, Andrew P. Jackson

**Affiliations:** 1 Medical Research Council Human Genetics Unit, MRC Institute of Genetics and Molecular Medicine, University of Edinburgh, Edinburgh EH4 2XU, UK; 2 Medical Research Council Mitochondrial Biology Unit, Cambridge CB2 0XY, UK

## Abstract

The presence of ribonucleotides in genomic DNA is undesirable given their increased susceptibility to hydrolysis. Ribonuclease (RNase) H enzymes that recognize and process such embedded ribonucleotides are present in all domains of life. However, in unicellular organisms such as budding yeast, they are not required for viability or even efficient cellular proliferation, while in humans, RNase H2 hypomorphic mutations cause the neuroinflammatory disorder Aicardi-Goutières syndrome. Here, we report that RNase H2 is an essential enzyme in mice, required for embryonic growth from gastrulation onward. RNase H2 null embryos accumulate large numbers of single (or di-) ribonucleotides embedded in their genomic DNA (>1,000,000 per cell), resulting in genome instability and a p53-dependent DNA-damage response. Our findings establish RNase H2 as a key mammalian genome surveillance enzyme required for ribonucleotide removal and demonstrate that ribonucleotides are the most commonly occurring endogenous nucleotide base lesion in replicating cells.

## Graphical abstract

**Graphical Abstract:**

**Figure:**
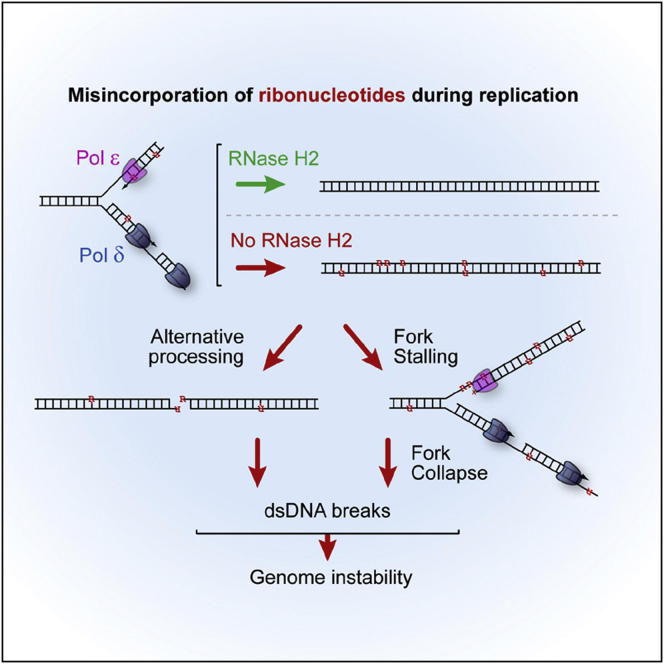

**Highlights:**

► Ribonucleotides are the most common nucleotide base lesion in the mouse genome ► RNase H2 is a key genome surveillance enzyme required for removal of nucleotides ► RNase H2 is essential for mammalian development ► Without RNase H2, cells exhibit genome instability and p53 pathway activation

## Teaser

DNA polymerases can incorporate more than a million ribonucleotides into replicating mouse DNA in each cell, making ribonucleotides the most abundant kind of DNA lesion. RNase H2, which removes this “damage,” has an essential role in genome surveillance and is required for embryonic development.

## Introduction

DNA is believed to have evolved from an ancestral RNA world as a more stable store of genetic information (Alberts et al., 2002; Cech, 2011). Ribonucleotides differ from deoxynucleotides by the presence of a single reactive hydroxyl group at the 2′ position of the ribose sugar, rendering RNA ∼100,000-fold more susceptible to spontaneous hydrolysis under physiological conditions (Li and Breaker, 1999). The presence of ribonucleotides in genomic DNA is therefore undesirable, as it renders DNA more sensitive to strand breakage. It has long been thought that such misincorporation is prevented by the stringent selectivity of replicative DNA polymerases, favoring deoxynucleoside triphosphate (dNTP) over ribonucleoside triphosphate (rNTP) substrates (Joyce, 1997). However, recent in vitro experiments have demonstrated that, under physiologically relevant conditions in which rNTPs substantially exceed dNTPs, such DNA polymerases may incorporate a ribonucleotide base every few thousand base pairs (Nick McElhinny et al., 2010a). Budding yeast expressing a less selective replicative polymerase only displayed widespread ribonucleotide incorporation when ribonuclease (RNase) H2 activity was genetically abolished (Nick McElhinny et al., 2010b). This directly implicated RNase H2 in the removal of such ribonucleotides.

RNase H enzymes hydrolyze the RNA strand of RNA/DNA hybrids (Stein and Hausen, 1969). Such hybrids form during many cellular processes, including DNA replication (Machida et al., 1977), telomere elongation (Förstemann and Lingner, 2005), and transcription (Huertas and Aguilera, 2003; Li and Manley, 2005). Eukaryotes have two types of RNase H with distinct biochemical properties and substrate specificity (reviewed in Cerritelli and Crouch, 2009). RNase H1 is a processive monomeric enzyme that requires interaction with 2′-OH groups from four consecutive ribonucleotides for efficient substrate cleavage (Nowotny et al., 2007). Mammalian RNase H1 has two isoforms: a nuclear isoform of undefined function and a mitochondrial isoform that is essential for mitochondrial DNA replication (Cerritelli et al., 2003). However, the predominant source of RNase H activity in mammalian cells is RNase H2 (Büsen, 1980). Like RNase H1, it digests the RNA strand of RNA/DNA hybrids in a processive manner (Chon et al., 2009), but it also recognizes single ribonucleotides in a DNA duplex and cleaves the 5′-phosphodiester bond of the ribonucleotide (Eder et al., 1993). In eukaryotes, RNase H2 is a multimeric complex consisting of three subunits: RNASEH2A, RNASEH2B, and RNASEH2C (Crow et al., 2006a; Jeong et al., 2004). The RNASEH2A subunit contains the catalytic center, whereas the closely intertwined auxiliary RNASEH2B and C subunits are likely involved in interactions with other proteins (Figiel et al., 2011; Reijns et al., 2011; Shaban et al., 2010). A PIP box motif at the C terminus of the RNASEH2B subunit guides the interaction between RNase H2 and PCNA (Chon et al., 2009) and its localization to replication foci (Bubeck et al., 2011), consistent with a role for the RNase H2 enzyme in DNA replication and/or repair.

Mutations in all three genes that encode the RNase H2 subunits cause the autosomal-recessive disorder Aicardi-Goutières syndrome (AGS) (Crow et al., 2006a). This early-onset neuroinflammatory condition mimics congenital viral infection and has immunological similarities to the autoimmune disease systemic lupus erythematosus (Ramantani et al., 2010). RNase H2 mutations that cause AGS result in partial rather than absolute loss of RNase H2 enzyme function (Reijns et al., 2011; Rice et al., 2007). Two further enzymes have been implicated in AGS: the 3′→5′ DNA exonuclease TREX1 (Crow et al., 2006b) and a dNTP triphosphohydrolase, SAMHD1 (Rice et al., 2009). Innate immune-mediated inflammation is thought to result from the accumulation of endogenous nucleic acid species that are usually degraded by these enzymes, e.g., during DNA replication/repair (Yang et al., 2007) or suppression of endogenous retroelements (Manel and Littman, 2011; Stetson et al., 2008).

The nucleic acids that may accumulate as a consequence of impaired RNase H2 function are yet to be defined, and although RNase H2 enzyme activity has been studied for more than 40 years (Stein and Hausen, 1969), its cellular functions are poorly understood. Initially, RNase H2 was proposed to act in removal of the RNA oligonucleotides that prime Okazaki fragment synthesis during lagging-strand replication. In vitro biochemical studies indicate that sequential action of RNase H2 and FEN1 are sufficient to complete this process (Goulian et al., 1990; Turchi et al., 1994). However, primer removal through flap processing by FEN1/DNA2 has become the predominant model for Okazaki fragment maturation (Burgers, 2009; Rossi and Bambara, 2006). RNase H2 may also be important for the resolution of R loops that arise during transcription (El Hage et al., 2010) or for the repression of endogenous retroelements (Cerritelli and Crouch, 2009). Finally, the distinctive property that allows RNase H2 to recognize and cleave single ribonucleotides that are embedded in DNA duplexes suggests a role in the removal of such nucleotides from genomic DNA (Rydberg and Game, 2002).

Here, we performed targeted mutagenesis of the mouse *Rnaseh2b* gene to gain insight into the in vivo role of the mammalian RNase H2 enzyme. Ablation of *Rnaseh2b* in mice leads to early embryonic lethality due to elevated DNA damage and reduced cellular proliferation during gastrulation. We establish that the growth arrest is the consequence of a p53-dependent DNA damage response associated with the accumulation of single ribonucleotides in genomic DNA. Thus, we demonstrate that removal of ribonucleotides to preserve genome integrity is an essential cellular function of RNase H2 in mammals.

## Results

### *Rnaseh2b* Is a Developmentally Essential Gene

**Figure 1:**
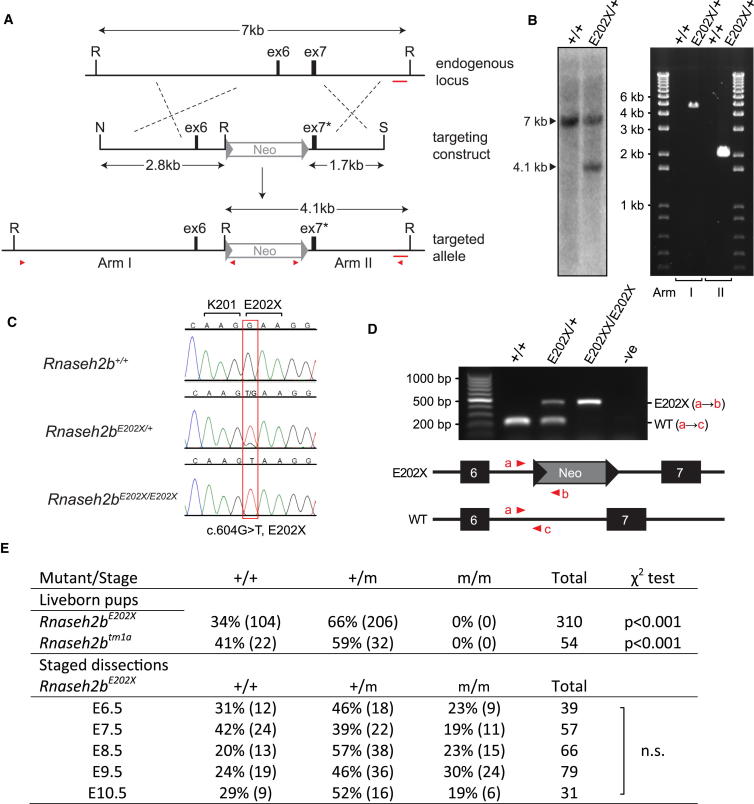
Targeted Inactivation of the *Rnaseh2b* Gene Causes Embryonic Lethality (A) Schematic depicting targeted mutagenesis of exon 7 of the *Rnaseh2b* gene. (Top) A 7 kb segment of the *Rnaseh2b* genomic locus; exons 6 (ex6) and 7 (ex7) are indicated by black boxes, flanked by EcoRI sites (R). (Middle) *Not*I (N)-SalI (S) restriction fragment of the final targeting construct, comprising 4.5 kb of genomic DNA and a Neomycin selection cassette (Neo) flanked by Cre recombinase loxP sites (triangles). (Bottom) Successfully targeted endogenous locus containing the mutagenized exon 7 (ex7^∗^). Red arrowheads indicate primers used to amplify arm I and arm II to confirm correct targeting. (B) Southern blotting and long-range PCR confirm successful targeting by homologous recombination. The introduction of an additional EcoRI site results in a restriction fragment of 4.1 kb detected on Southern blotting with the 400 bp probe (red bar in A) for the targeted ES cells (E202X/+) that is not present in parental DNA (+/+). Arm I (4.7 kb) and arm II (2.2 kb) fragments are amplified by PCR in correctly targeted ES cells only. (C) Sequencing traces for *Rnaseh2b^+/+^*, *Rnaseh2b^E202X/+^*, and *Rnaseh2^E202X/E202X^* DNA show the introduced nonsense mutation (red box). (D) Multiplex PCR for mouse genotyping. (Top) A 221 bp PCR product (a→c) is present in wild-type mice (+/+); mice containing the *Rnaseh2b^E202X^* allele (also) give a 460 bp product (a→b). (Bottom) Schematic indicating position of forward (a) and reverse primers (b and c). (E) Mice with null mutations (m) in *Rnaseh2b* are not postnatally viable, whereas E6.5–E10.5 embryos are present at Mendelian ratios. Genotype frequencies for offspring at weaning (and for embryos at indicated stages) derived from *Rnaseh2b^E202X/+^* and *RNaseh2b^tm1a/+^* intercrosses respectively. p values, χ^2^ test; n.s., not significant; m, mutant allele. *Rnaseh2b* accession number: NM_026001.2.

*Rnaseh2b^E202X^* embryonic stem (ES) cells with a premature stop codon in exon 7 of *Rnaseh2b* were generated by targeted homologous recombination (Figure 1A). Correct recombination of both arms of the targeting cassette was confirmed by Southern blotting and long-range PCR (Figures 1A and 1B) and the presence of a nonsense mutation at codon 202 (E202X) established by sequencing (Figure 1C). ES cells were injected into C57BL/6J host blastocysts to generate germline chimeras and subsequently heterozygous *Rnaseh2b^E202X/+^* mice. Intercrossing of *Rnaseh2b^E202X/+^* animals failed to yield live-born homozygous null mice (p < 0.001; Figures 1D and 1E). Similarly, homozygotes were not present (p < 0.001) in litters of a second independent line, *Rnaseh2b^tm1a^*, derived from EUCOMM “knockout-first” ES cells.

**Figure 2:**
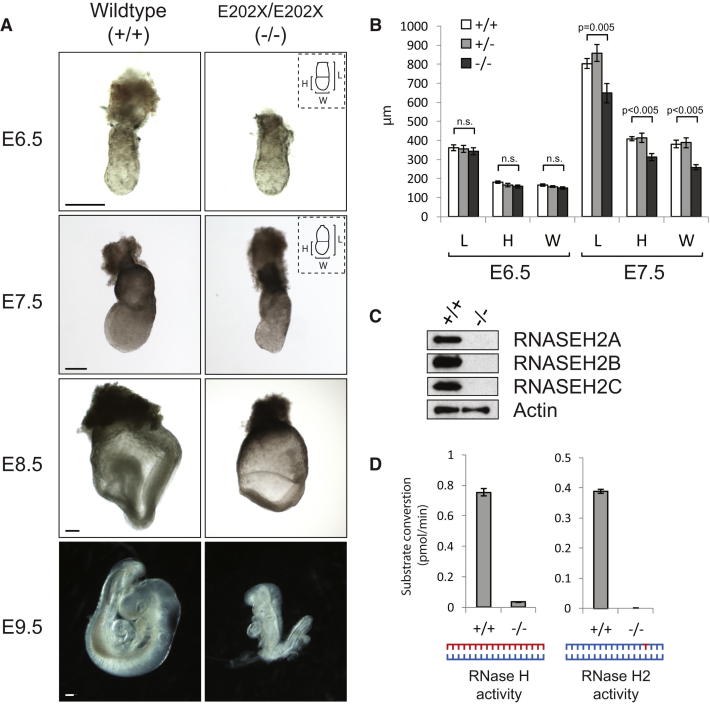
*Rnaseh2b^E202X/E202X^* Embryos Exhibit Severe Growth Failure during Early Development, Resulting in Embryonic Lethality (A and B) Growth failure in mutant embryos starts at gastrulation. (A) Photomicrographs of representative embryos from embryonic stages E6.5, E7.5, E8.5, and E9.5. Scale bars, 200 μm. (B) There is a significant difference in length (L), width (W), and height (H) between wild-type (+/+) and mutant *Rnaseh2b^E202X/E202X^* embryos (denoted as *−/−*) at E7.5, but not at E6.5. (E7.5, 7 litters: n = 22,21,11; E6.5, 6 litters: n = 12,18,9 for +/+, +/−, and −/−, respectively). Error bars represent SEM; t test; n.s., not significant. (C) Immunoblotting demonstrates that all three RNase H2 subunits (RNASEH2A, B, and C) are absent from mutant embryo lysates. Loading control, actin. (D) Type 2 RNase H activity is undetectable in mutant embryos, and total cellular RNase H activity is reduced to < 10%. Cleavage of RNase H (RNA/DNA hybrid) and RNase H2-specific substrates by mutant and wild-type E9.5 embryo lysates was measured using fluorescence-based assays. Error bars represent SD. n = 3 replicates. See also Figure S1.

As no viable homozygous animals were obtained from either of these alleles, we concluded that *Rnaseh2b* was likely to be an essential gene that is required for embryonic viability. At embryonic day 6.5 (E6.5), *Rnaseh2b^E202X/E202X^* embryos were present at Mendelian ratios and were almost indistinguishable in size from wild-type littermates, suggesting normal progression of early embryogenesis (Figures 2A and 2B). However, by E7.5, there was a 23% and 32% decrease in embryonic height and width, respectively (p < 0.005; Figure 2B), suggesting a failure to increase the rate of proliferation in the epiblast that normally occurs at the onset of gastrulation (Mac Auley et al., 1993; Snow, 1977). Though all mutants proceeded through gastrulation, the embryos remained reduced in size and were developmentally retarded (Figure 2A). By E9.5 (n = 23), they were frequently truncated (57%) with few or very small postcervical somites (52%) and defects in allantois development (48%). At E10.5, histology demonstrated increased numbers of cells with condensed or fragmented nuclei, and at E11.5, a terminal phenotype was evident with loss of tissue morphology and integrity (data not shown).

### *Rnaseh2b^E202X^* Results in Absolute Loss of RNase H2 Complex Function

**Figure S1:**
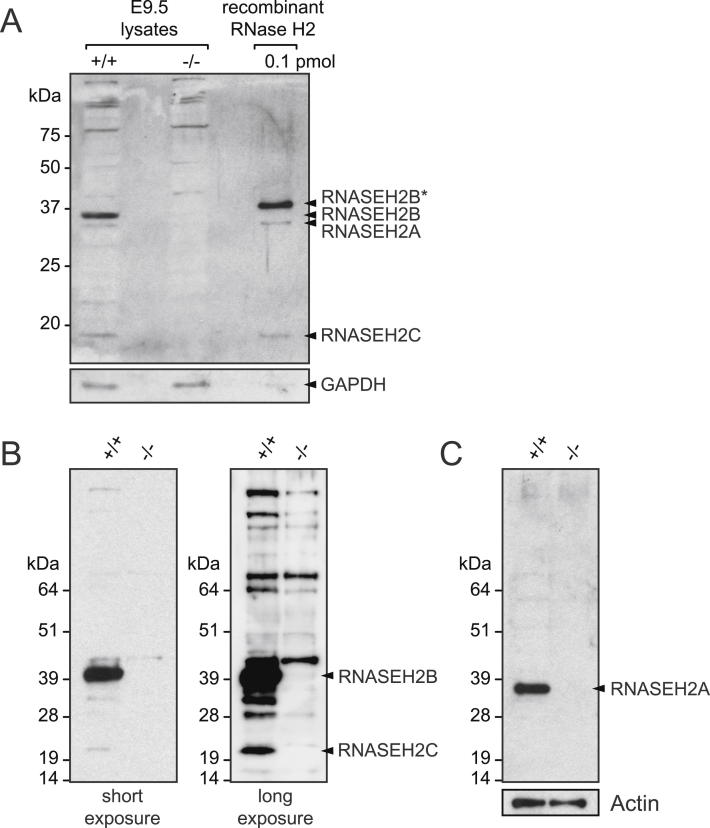
Full-Length Images of Immunoblots Probed with α-Rnase H2 Antibodies, Related to Figure 2 (A) Affinity purified antibodies raised against recombinant mouse RNase H2 detect RNASEH2A, RNASEH2B and RNASEH2C by immunoblotting. Lysates from wild-type (+/+) and *Rnaseh2b^E202X/E202X^* (−/−) E9.5 embryos were separated by SDS-PAGE alongside 0.1 pmol of purified recombinant mouse RNase H2. Recombinant RNASEH2B (RNASEH2B^∗^) migrates more slowly due to the presence of linker sequence. Loading control, GAPDH. (B and C) Full length immunoblots corresponding to those presented in Figure 2C. (B) RNASEH2B and RNASEH2C are detected in wild-type, but not in null embryo lysates. A long exposure image is provided to show that low levels of a truncated RNASEH2B^E202X^ (predicted molecular weight, 23 kDa) are not apparent in RNaseh2^null^ embryo lysates. (C) RNASEH2A was detected on the same blot, using a commercial anti-RNASEH2A antibody (Origene). Actin loading control shown in panel C also applies to the blots in panels B.

To examine the effect of the *Rnaseh2b^E202X^* mutation on the RNase H2 enzyme, immunoblotting was performed with affinity-purified antibodies. As the premature stop codon is not in the final *Rnaseh2b* exon, it is expected to cause nonsense-mediated decay of the transcript. Consistent with this, we did not detect a truncated RNASEH2B^E202X^ protein (Figure S1B available online). Also, full-length RNASEH2A, B, and C subunits were all undetectable in E9.5 *Rnaseh2b^E202X/E202X^* embryo lysates (Figure 2C), indicating that RNASEH2B is required for in vivo stability of the heterotrimeric complex. Furthermore, type 2 RNase H activity against a DNA duplex oligonucleotide with an embedded ribonucleotide was undetectable in *Rnaseh2b^E202X/E202X^* embryos (Figure 2D). In contrast, general RNase H activity was retained, albeit at <10% of wild-type embryo levels (Figure 2D), consistent with retention of normal RNase H1 activity. Given that RNase H2 is absent from *Rnaseh2b^E202X/E202X^* embryos, we hereafter refer to these embryos and their derivative cells as RNaseH2^null^ and their genotype as *Rnaseh2b^−/−^*. Significantly, such embryos entirely lack detectable enzyme activity against isolated ribonucleotides embedded in DNA.

### RNase H2 Is Highly Expressed in Proliferating Cells

**Figure 3:**
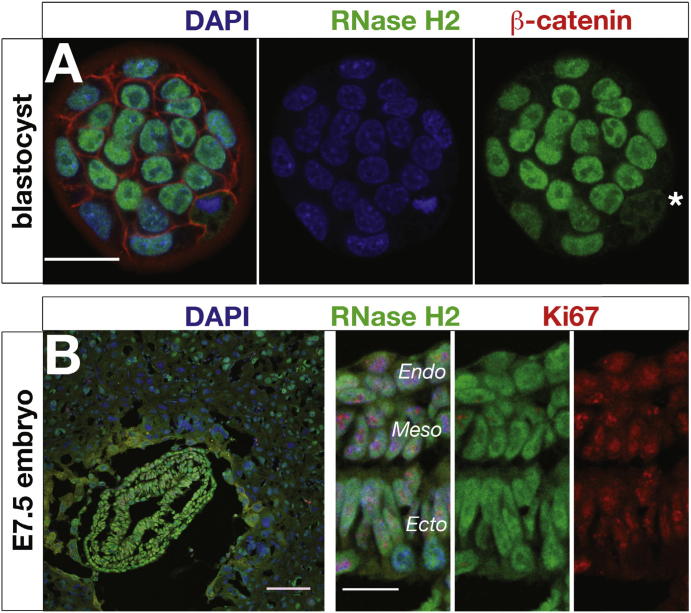
The RNase H2 Enzyme Is Expressed in Actively Proliferating Cells from Early Embryogenesis (A) RNase H2 is expressed from early embryogenesis. Whole-mount immunostaining of wild-type mouse blastocysts detects endogenous RNase H2 expression in the nucleus of interphase cells and dispersed throughout the cell at mitosis (^∗^). Scale bar, 20 μm. (B) RNase H2 is expressed in all three cell layers of gastrulating mouse embryos. Confocal image of a wild-type cryosectioned E7.5 embryo, with Ki67 marking actively proliferating cells. Scale bar, 100 μm. (Insert) Higher-power view demonstrates strong nuclear localization in all three embryonic layers: endoderm (Endo), mesoderm (Meso), and ectoderm (Ecto). Scale bar, 20 μm. See also Figure S2.

**Figure S2:**
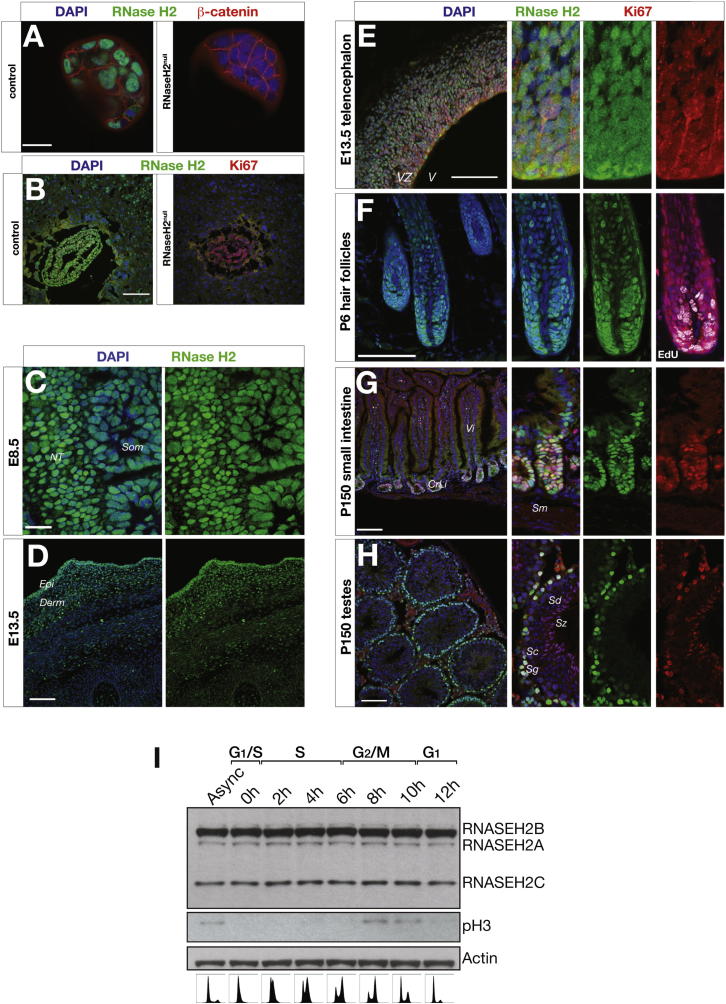
RNaseH2 Is Expressed in Actively Proliferating Cells during Development and Postnatally, Related to Figure 3 (A and B) RNase H2 expression is absent from RNaseH2^null^ embryos as shown by whole-mount immunostaining of (A) blastocyst stage (E3.5) and (B) gastrulation stage (E7.5) embryos. Control embryo images were captured using the same microscopy settings as mutants. The panel from Figure 3B of an E7.5 control embryo is shown here for comparison. (C and D) RNase H2 expression is widespread in early embryos, but becomes more restricted as development advances. (C) RNase H2 remains ubiquitously expressed at E8.5. Somites (Som) and neural tube (NT). (D) However, by E13.5, higher level expression becomes restricted: widespread expression is seen in the dorsal epidermis (Epi) and dermis (Derm), whereas expression in underlying skeletal muscle and cartilaginous structures is greatly reduced. (E-H) RNase H2 is maintained in proliferative cell populations in the embryo and adult, and correlates with Ki67 expression. (E) Sagittal cryosections of dorsal telencephalon during neurogenesis at E13.5. The ventricular zone (VZ) and subventricular zone contain proliferating progenitor cells and recently generated neurons. Ventricle (V). (F) RNase H2 expression in P6 juvenile dorsal skin is localized to proliferating epidermal progenitors in anagen hair follicles, and the basal layer of interfollicular epidermis (data not shown). The rightmost panel additionally shows S-phase cells (white) marked by pulse labeling with EdU in a separate hair follicle. (G) In adult intestinal endothelium, RNase H2 expression also correlates with sites of proliferation, and is present in the crypts of Lieberkühn (CrLi), between the villi of the mucosa of the small intestine of a 5 month old adult mouse, while it is absent from the underlying submucosa (Sm). (H) RNase H2 is also expressed in the outer edges of seminiferous tubules containing cells undergoing spermatogenesis. RNase H2 is expressed in the least mature cells (spermatogonias or sperm stem cells, Sg) located at the base of the epithelium, with low levels of expression in more matured cells spermatocytes (Sc). No expression is detected in more differentiated spermatids (Sd) or the most mature cells, the spermatozoa (Sz), which are released in the lumen. Additionally, expression of RNase H2 is present at other sites with life-long proliferative populations, such as the stratum basale of the eosophagus, the thymus cortex and the spleen red pulp (the site of life-long hematopoiesis in the mouse) and germinal centers of the white pulp (data not shown). Scale bars A,C: 20 μm; F: 50 μm; B,D,E,G,H: 100 μm. (I) The RNase H2 enzyme complex is present at constant levels throughout the cell cycle. Total cell extracts from synchronized HeLa cells, harvested at indicated time points after release from 2 mM double thymidine block. Immunoblotting demonstrates constant levels of RNase H2 subunits at all time points. Loading control, actin. Bottom: Propidium Iodide FACS analysis to determine cell cycle stages. Probing with anti-phospho-histone H3 antibody (pH3) defines mitosis.

The anti-RNase H2 antibody specifically detected RNase H2 when used for immunofluorescence microscopy, as staining was absent in null embryos (Figures S2A and S2B). Endogenous RNase H2 exhibited nuclear localization in early embryos, consistent with proposed roles in DNA replication and repair. Expression was observed in blastocysts (Figure 3A) and in all three embryonic layers during gastrulation (Figure 3B), reflecting a ubiquitous presence at early stages of development. Later in embryogenesis and postnatally, expression became more restricted to highly proliferative tissues, such as the subventricular zone during neurogenesis and perinatal hair follicles (Figures S2C–S2F). In adults, RNase H2 was present in proliferative tissues, including intestinal crypt epithelium, and testes (Figures S2G and S2H). Expression correlated most closely with the proliferation marker Ki67, suggesting that RNase H2 is preferentially expressed in actively cycling cells at all stages of the cell cycle. In support of this, western analysis from synchronized HeLa cells demonstrated uniform expression levels of all three RNase H2 subunits throughout the cell cycle (Figure S2I).

### A p53-Dependent Damage Response Is Evident in RNaseH2^null^ Embryos

**Figure 4:**
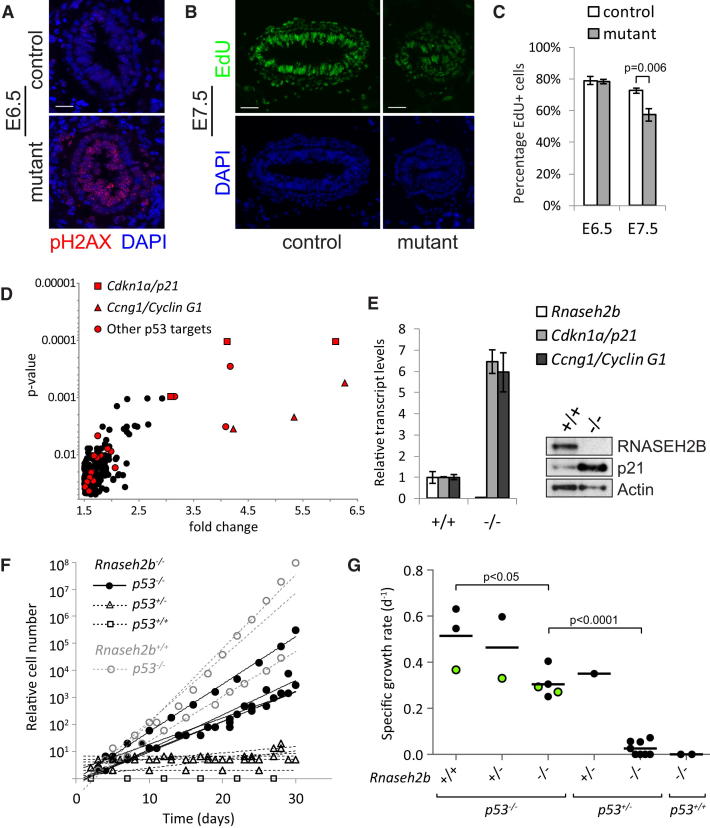
A p53-Dependent DNA-Damage Response Is Activated in RNase H2-Deficient Embryos, Leading to Arrest in Cellular Proliferation (A) Markedly elevated levels of nuclear pH2AX foci are evident in the epiblast of E6.5 embryos. Confocal projection of transverse cryosections through the decidua of E6.5 mutant RNaseH2^null^ and control littermate embryos. Scale bar, 10 μm. (B and C) The number of S phase epiblast cells is reduced in E7.5 embryos. (B) Representative immunofluorescence confocal projections of 10 μm cryosections from E7.5 mutant and control embryos. Embryos fixed 1 hr after intraperitoneal injection of 100 mg/kg into pregnant females and EdU visualized by Click-iT (Invitrogen), counterstained with DAPI (blue). Scale bar, 50 μm. (C) Relative proportions of EdU- incorporating cells in E6.5 and E7.5 embryos demonstrate a significant reduction at E7.5 in RNaseH2^null^ embryos (t test; n = 5 embryos per data point, >200 epiblast cells/embryo). S phase index determined from EdU-positive nuclei/total nuclei. Error bars represent SEM. (D) Significant upregulation of p53 target genes in E9.5 RNaseH2^null^ embryos is detected by Illumina MouseWG-6 v2.0 Expression BeadChip microarray analysis. Plotted data points correspond to Illumina probes. (E) qPCR confirms a 6-fold upregulation of *Cyclin G1* and *p21* transcripts in E9.5 mutant embryos (error bars represent SD of technical triplicates). Immunoblotting of total cell lysates from E9.5 mutant embryos demonstrates increased p21 protein levels. Loading control, actin. (F and G) Cell-cycle arrest in RNaseH2^null^ embryos is p53 dependent. (F) Growth kinetics from primary culture of mesenchymal cells recovered from E10.5 and E11.5 RNaseH2^null^ embryos show that RNaseH2^null^ cells from *p53^−/−^* (n = 5), but not *p53^+/−^* (n = 8) and *p53^+/+^* (n = 2), littermates are capable of proliferation. Growth curves for *Rnase2b^+/+^;p53^−/−^* cells (n = 3) derived from littermates are shown for comparison. (G) Specific growth rates of MEFs calculated from growth kinetics as shown in (F). *Rnase2b^−/−^;*p53^−/−^ cells initially grew with doubling times of 2.4 ± 0.4 days compared with *Rnase2b^+/+^;*p53^−/−^ cells that doubled every 1.5 ± 0.5 days (p < 0.05). This difference became negligible at later passages (data not shown). Green circles correspond to MEF lines used for further analysis. See also Figure S3.

RNaseH2^null^ embryonic growth failure could be the consequence of inefficient DNA replication or activated DNA damage signaling. Embryonic tissues were therefore immunostained for histone H2A.X phosphorylated at serine 139 (pH2AX), a marker of DNA-damage response to double-strand breaks and arrested replication (Rogakou et al., 1998; Ward and Chen, 2001). Though no difference in pH2AX levels was observed between mutant and control blastocysts, at E6.5, there was a substantial increase in nuclear pH2AX staining in epiblast cells of RNaseH2^null^ embryos (Figure 4A), coinciding with a period of rapid cell cycles of less than 6 hr (Snow, 1977).

At E6.5, there was no alteration in the percentage of epiblast cells undergoing active DNA synthesis; however, by E7.5, a significant reduction was evident (Figures 4B and 4C). Reduced embryo growth appeared to be the result of arrested cell proliferation, rather than cell death, as no widespread increase in apoptosis was observed at E7.5 or E9.5 by activated caspase 3 immunostaining (data not shown).

To investigate the molecular basis of this growth arrest, whole-genome expression analysis was performed using Illumina microarrays. Transcript levels of 197 genes were significantly upregulated in E9.5 mutant embryos, whereas 115 genes were downregulated when compared to age-matched controls (p < 0.05; > 1.5-fold change). Of note, the four genes with the greatest fold increase in expression (Figure 4D), *Ccng1/Cyclin G1*, *Cdkn1a/p21*, *Phlda3*, and *Trp53inp1*, were all targets of the p53 transcriptional activator, a key transducer of ATM/ATR DNA damage signaling (Tibbetts et al., 1999; Yajima et al., 2006). Increased *Cyclin G1* and *p21* expression was confirmed by qPCR and immunoblotting (Figure 4E). In addition, *Rnaseh2b* was found to be <0.02% of wild-type by qPCR, in keeping with nonsense-mediated decay of this transcript (Figure 4E). Although RNase H2 could have a role in suppressing expression of endogenous retroelements (Bhoj and Chen, 2008), no changes in retroelement transcript levels were identified, indicating that there was no widespread dysregulation of retroelements at this stage of embryonic development. Similarly, there was no transcriptional evidence of an immune-mediated process (data not shown).

**Figure S3:**
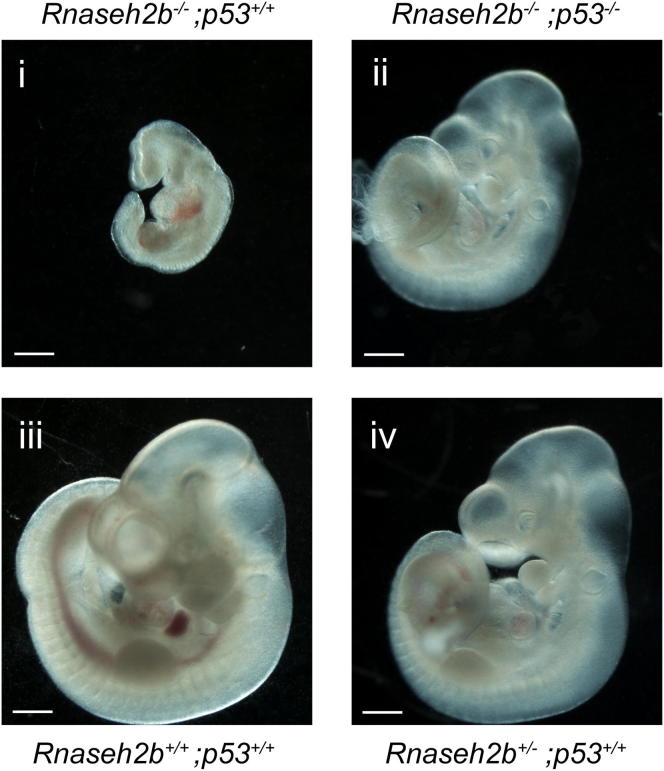
Partial Rescue of the RNaseH2^null^ Mutant Phenotype in a *p53^−/−^* Background, Related to Figure 4 Embryo size was substantially rescued in all embryos analyzed at E9.5 (7/7) relative to previously analyzed RNaseH2^null^ embryos. At E10.5, 3 out of 4 *Rnaseh2b^−/−^;p53^−/−^* embryos were also much larger than *Rnaseh2b^−/−^;p53^+/+^* mutants but not as large as littermate controls. Measurement of E9.5 embryos confirmed that growth was not completely rescued: crown-rump length *Rnaseh2b^−/−^;p53^−/−^* 1.76 ± 0.44 mm (SD; n = 7), *Rnaseh2b^+/^;p53^−/−^* 2.33 ± 0.47 mm (SD; n = 10; t test, p = 0.02). Images shown: E10.5 (i) *Rnaseh2b^−/−^*;*p53^+/+^* (ii) *Rnaseh2b^−/−^*;*p53^−/−^* (iii, iv) littermate control embryos for i and ii respectively (scale bars, 500 μm). A morphological rescue of the RNaseH2^null^ phenotype was observed at E9.5 and E10.5: *Rnaseh2b^−/−^;p53^−/−^* mutants were not developmentally retarded and did not exhibit truncation, somite or allantoic defects (n = 11). Total embryos analyzed: E9.5 n = 33; E10.5 n = 66. The previously described partially penetrant embryonic *p53^−/−^* phenotype was present in some *p53^−/−^* embryos with defects in neural-tube closure evident (Armstrong et al., 1995; Sah et al., 1995). Some background abnormalities were also evident in *Rnaseh2b^+/^;p53^+/^* control littermate embryos (e.g., 11% exhibited severe runting at E10.5). Given the partial phenotypic rescue in growth, along with the marked genome instability observed in *Rnaseh2b^−/−^;p53^−/−^* MEFs, it would appear unlikely that these double mutant embryos will be viable at later embryonic stages, as has also been the case for previously reported DNA damage mutants crossed onto a *p53^−/−^* background (Adam et al., 2007; Bouwman et al., 2011; Hakem et al., 1997).

We postulated that the DNA damage observed in the E6.5 embryos led to p53 activation and reduced cellular proliferation as a consequence of cyclin G1 and p21-mediated cell-cycle arrest (Cazzalini et al., 2010; Kimura et al., 2001). In keeping with this, partial rescue of the RNaseH2^null^ embryonic phenotype was observed in a *p53^−/−^* background (Figure S3). Loss of p53 also fully rescued defective cell proliferation (Figure 4F) in primary cultures of E10.5/E11.5 mesodermal tissue, performed to generate mouse embryonic fibroblast (MEF) cell lines. *Rnaseh2b^−/−^;p53^+/+^* cells completely failed to proliferate in culture (2/2), whereas those derived from *Rnaseh2b^−/−^;p53^−/−^* embryos grew well (p < 0.001; 5/5), though at 64% of the rate of *Rnaseh2b^+/+^;p53^−/−^* MEFs (Figures 4F and 4G). *Rnaseh2b^−/−^;p53^+/−^* cells showed very limited growth, and though MEF cell lines were eventually established from three out of eight embryos, all were found to have then lost the remaining wild-type *p53* allele (data not shown). In conclusion, loss of RNase H2 enzyme activity results in p53-mediated arrest of cell growth.

### Ribonucleotide Accumulation in Genomic DNA of RNaseH2^null^ Cells

**Figure 5:**
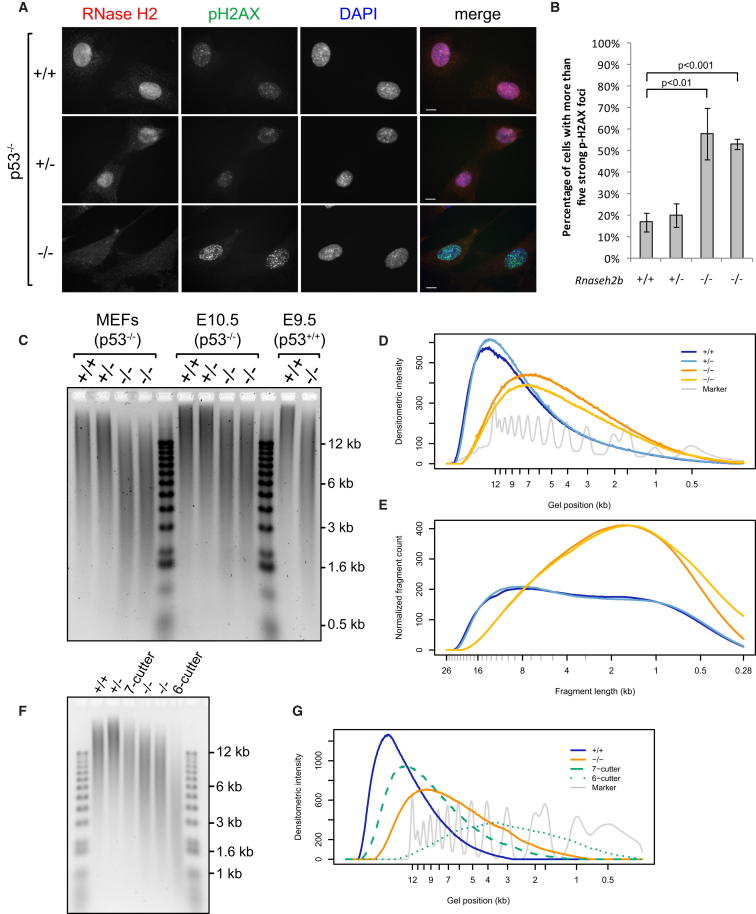
RNaseH2^null^ Genomic DNA Is More Sensitive to Alkali Hydrolysis (A and B) Levels of pH2AX foci are elevated in *Rnase2b^−/−^;p53^−/−^* MEFs. (A) Representative immunofluorescent images of *Rnaseh2b^+/+^*, *^+/−^*, *^−/−^* cell lines. Scale bar, 10 μm. (B) Quantification for (A). Cells with five or more strong pH2AX foci. Error bars represent SD. n = 3 expts, > 100 cells/expt, t test. From here on +/+, +/−, −/−, and −/− indicate *Rnaseh2b* genotypes of four independent MEF lines. (C and D) Genomic DNA from RNaseH2^null^ cells display markedly increased alkali sensitivity. (C) Representative gel of total nucleic acids from *Rnaseh2b^+/+^*, *^+/−^*, *^−/−^* MEFs and yolk sacs separated by alkaline agarose gel electrophoresis after alkaline hydrolysis. (D) Densitometry of the first five lanes of (C), plotted using Aida 2D densitometry software, demonstrates a substantial shift in migration of RNaseH2^null^ MEF genomic DNA fragments. (E) Quantification of DNA fragmentation pattern calculated from densitometry traces shown in (D). Densitometry intensity distribution is divided by the fragment length distribution to quantitate the proportion of molecules of a particular fragment size. Fragment counts are normalized so that total nucleotide number is equal between samples. (F and G) Alkali treatment fragments RNaseH2^null^ DNA to an average size that lies between 3.7 and 11 kb. (F) Fragmentation pattern of RNaseH2^null^ (−/−) alkali-treated DNA compared with that of the nicking endonucleases Nt.*Bsp*QI, which cuts mouse genomic DNA on average every 11 kb (7-cutter), and Nb.*Bts*I, which cuts on average every 3.7 kb (6-cutter). (G) Densitometry of selected lanes from (F). See also Figure S4.

**Figure S4:**
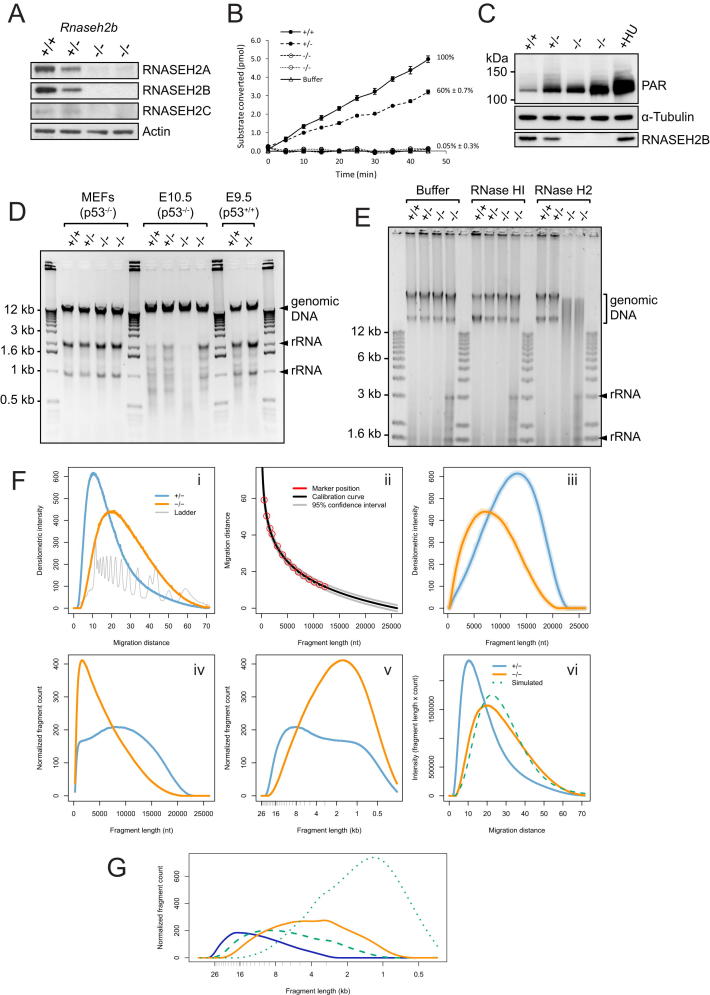
RNase H2 Is Absent from *Rnaseh2b^−/−^;p53^−/−^* MEFs; Quantitative Estimation of Ribonucleotide Levels in Genomic DNA, Related to Figure 5 (A) All three RNase H2 subunits are absent from MEFs derived from *Rnaseh2b^−/−^;p53^−/−^* embryos, as shown by immunoblotting. Loading control, Actin. (B) Whereas RNase H2 activity is reduced in *Rnaseh2b^+/−^;p53^−/−^* MEFs (+/−), substrate cleavage is not detected in two independent *Rnaseh2b^−/−^;p53^−/−^* lines (−/−), within the sensitivity of the assay. Error bars represent SEM of technical triplicates. (C) PolyADPribosylation is increased in RNaseH2^null^ MEFs. Immunoblot probed with α-PAR antibody. PolyADPribosylation occurs on a large number of proteins in response to DNA damage, but auto-PARylation of PARP1 predominates (Satoh and Lindahl, 1992). ‘+/+, +/−, −/− and −/−’ correspond to 4 independent MEF cell lines. ‘+HU’, *RNaseh2b^+/+^* MEFs treated with 300 μM hydroxyurea for 48 hr as a positive control. Immunoblot representative of 4 independent experiments. Densitometry of immunoblots confirmed that compared to the +/+ control polyADPribosylation was significantly increased in both −/− lines (n = 4, paired t test, p < 0.05 and p = 0.001 respectively for −/− lines; for the ‘+/−’ line, the p-value was not significant). (D) Untreated total nucleic acids purified from MEFs and yolk sacks (E9.5 or E10.5) separated by agarose gel electrophoresis. (E) Genomic DNA from E9.5 RNaseH2^null^ yolk sacks is cleaved by RNase H2, but not RNase HI, showing accumulation of incorporated mono or di-ribonucleotides. Treated nucleic acids were denatured in 90% formamide and separated by agarose gel electrophoresis. (F) Quantification of ribonucleotide incorporation rates: estimation by analytical methodology and simulation studies. (i-iii) Gel background intensity was uniformly subtracted from all raw densitometry distributions of electrophoresed alkali-digested genomic DNA (i). The distributions were transformed to nucleotide size (iii) on the basis of a DNA calibration curve derived from size standard ladders (ii) and a spline with 40 degrees of freedom used to produce a smoothed distribution. (iv) Analytical estimate: Histograms of fragment counts per nucleotide length were fitted to the smoothed distributions. These were modeled on the basis of a linear relationship between staining intensity and nucleotide content. Nucleotide number was scaled to 10^9^ per distribution. From these resulting histograms (iv) an analytical estimate for additional fragmentation in mutant (‘−/−’, orange) versus control DNA (here, ‘+/−’, blue) was obtained, by subtracting fragment count of control from that of mutant. Overall, this resulted in an estimate of 1 ribonucleotide every 7,600 nucleotides in RNaseH2^null^ MEFs (1 in 7.6 ± 0.8 kb; SD, n = 4, biological replicates compared to +/+ and +/− controls). Transforming the *x* axis to a log scale (v) allows the fragment count distributions to be viewed as areas under the curve, the excess area of the −/− relative to the +/− curve is proportional to the number of additional fragments the *Rnaseh2b*^−/−^ genome is hydrolyzed into, as also shown in Figures 5E and 6F. (vi) To explore the spatial distribution of incorporated ribonucleotides, a simulation study was performed in which the control distribution (solid blue) was transformed by random fragmentation, and a best-fit (dotted green) to the mutant distribution (solid orange) obtained. This simulation was broadly consistent with cleavage at random positions, however given that the fit is not exact it remains conceivable that ribonucleotide incorporation is in part non-random. The random cut simulation provided a frequency estimate of 1 in 6,100 nucleotides. The analytic estimate of 1 in 7.6 ± 0.8 kb (SD) is supported by experimental results that showed a site frequency between 1 in 3.7 and 11 kb (Figure 5F, G and Figure S4H) and a similar simulation result. As the mouse haploid genome contains ∼2.5x10^9^ bp (Waterston et al., 2002), each diploid nucleus contains 10^10^ nucleotides of DNA. Thus with a ribonucleotide frequency of 1 in 7.6 kb; in excess of 1.3 million ribonucleotide sites would be expected per replicating cell in the absence of RNase H2 activity. (G) Quantification of DNA fragmentation pattern calculated from densitometry traces shown in Figure 5G. Fragment counts normalized so that total nucleotide number is equal between samples.

*Rnaseh2b^−/−^;p53^−/−^* MEFs were next used to investigate the molecular consequences of RNase H2 loss. As expected, type 2 RNase H activity and the RNase H2 protein complex were absent in these cell lines (Figures S4A and S4B). Significantly elevated levels of pH2AX foci were also present (Figures 5A and 5B), indicative of DNA double-strand breaks and/or replication fork arrest. In addition, we examined polyADPribosylation (PAR) as another early marker of DNA-damage activation, which occurs in response to DNA breaks (Caldecott, 2008; Satoh and Lindahl, 1992). Levels of PAR were substantially raised in RNaseH2^null^ MEFs, as shown by immunoblotting (Figure S4C), confirming the presence of cellular DNA-strand breaks.

**Figure 6:**
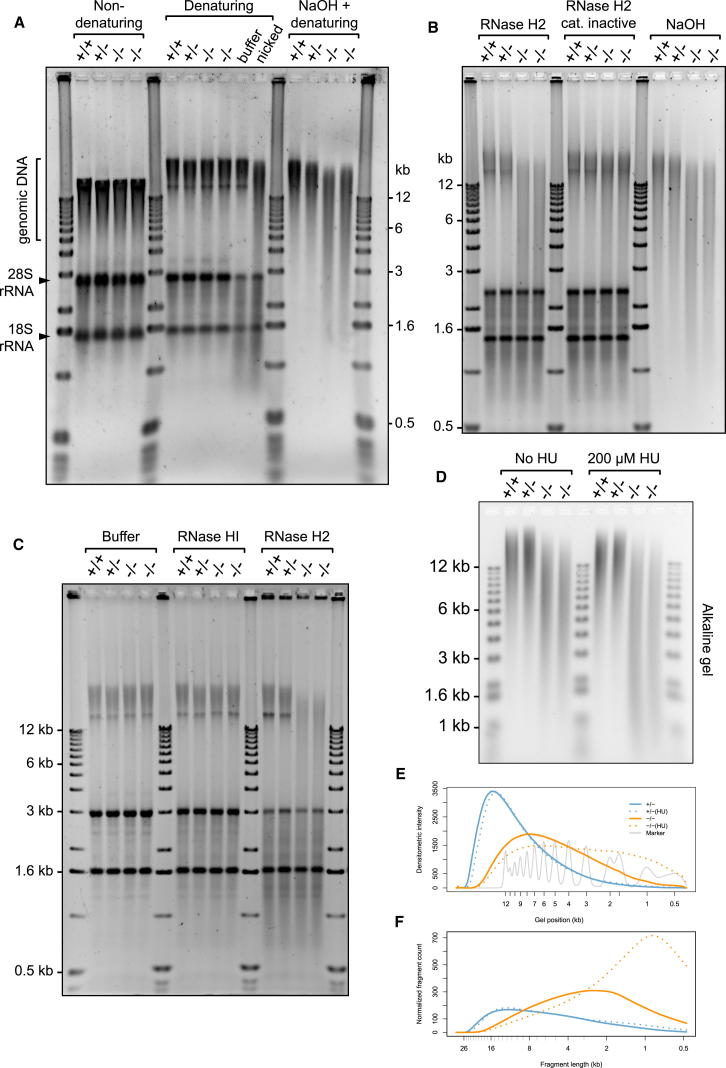
Covalently Incorporated Ribonucleotides Are Present in Nuclear DNA of RNaseH2^null^ Cells (A–C) *Rnase2b^−/−^;p53^−/−^* genomic DNA contains mono or diribonucleotides. Total nucleic acids isolated from *p53^−/−^* MEFs were separated by agarose gel electrophoresis under native conditions or after denaturation with 90% formamide. (A) Genomic DNA from RNaseH2^null^ cells does not contain elevated numbers of nicks. Increased nicking is detected only in genomic DNA treated with Nt.*Bsp*QI nicking endonuclease. (+/+, +/−, −/−,−/−) *Rnaseh2b* genotypes of four independent MEF lines. (B) RNase H2 fragments genomic DNA from RNaseH2^null^ cells to the same extent as hydrolysis with NaOH. Total nucleic acids isolated from passage matched *p53^−/−^* MEFs ± *Rnaseh2b* treated with purified recombinant human RNase H2, catalytically inactive RNase H2 (RNASEH2A-D34A/D169A), or NaOH and were then denatured with 90% formamide. (C) Recombinant RNase HI, which cleaves DNA duplexes with three or more embedded ribonucleotides, does not fragment DNA from RNaseH2^null^ MEFs. Treated nucleic acids were denatured with 90% formamide. (D–F) RNaseH2^null^ cells have increased ribonucleotide incorporation, reflected by enhanced alkali sensitivity after low-dose hydroxyurea treatment. Alkali gel electrophoresis of total nucleic acids from four independent MEF cell lines with and without hydroxyuea (HU) treatment (200 μM for 48 hr). (E) Densitometry traces of selected lanes from (D) as indicated and (F) quantification of fragmentation pattern. See also Figure S5.

DNA damage in RNaseH2^null^ cells might arise as the consequence of undegraded Okazaki RNA primers, misincorporation of ribonucleotides by DNA polymerases, or transcriptionally induced R loops. To discriminate between these possibilities, total nucleic acids from the MEFs were separated by gel electrophoresis under alkaline conditions. Substantially increased mobility of genomic DNA from both RNaseH2^null^ cell lines after treatment with alkali was observed relative to control genomic DNA (Figures 5C–5E), whereas no significant difference was evident upon electrophoresis under neutral conditions (Figure S4D). Given that phosphodiester bonds 3′ of ribonucleotides, but not deoxynucleotides, are sensitive to alkali hydrolysis through nucleophilic attack by the 2′ hydroxyl group (Lipkin et al., 1954), such increased fragmentation (termed alkali sensitivity) likely indicates the incorporation of ribonucleotides into genomic DNA. Given that alkali treatment also denatures DNA, increased electrophoretic mobility could also be the consequence of increased nicking, or gaps, in genomic DNA. To address this possibility, gel electrophoresis of total cellular nucleic acids was performed after formamide denaturation, which denatures DNA (Tang et al., 1989) without hydrolyzing ribonucleotide phosphodiester bonds (Figure 6A). Under these conditions, RNaseH2^null^ DNA did not demonstrate increased mobility, in contrast to DNA treated with Nt.*Bsp*QI nicking endonuclease, which on average introduces nicks every ∼11 kb. Therefore, the observed alkali-sensitive sites were consistent with covalently incorporated ribonucleotides, rather than gaps or nicks in genomic DNA.

**Figure S5:**
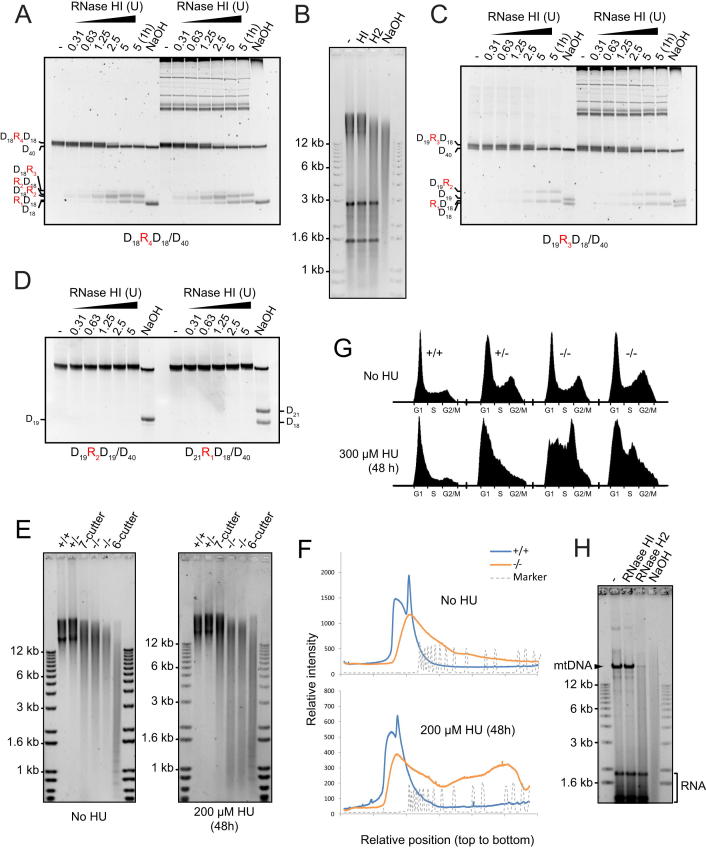
*E. coli* RNase HI Cleaves Substrates with Three or More Embedded Ribonucleotides; Increased Ribonucleotide Incorporation after Hydroxyurea Treatment, Related to Figure 6 (A) Substrate (5 pmol) with 4 ribonucleotides embedded in a 40 bp DNA duplex (D_18_R_4_D_18_/D_40_) was incubated for 5 min (or 1 hr where indicated) at 37°C with *E. coli* RNase HI (NEB) in the absence or presence of 0.5 μg of RNaseH2^null^ nucleic acids, and separated by denaturing PAGE. (B) RNaseH2^null^ genomic DNA in the same reactions as panel A (1 hr incubations), is not cleaved by RNase HI, but is hydrolyzed by RNase H2 or alkaline treatment (agarose gel electrophoresis after formamide denaturation to resolve high molecular weight nucleic acids). (C) As panel A: a substrate with 3 embedded ribonucleotides (D_19_R_3_D_18_/D_40_) is completely cleaved by RNase HI. (D) Substrates with 2 (D_19_R_2_D_19_/D_40_) or 1 (D_21_R_1_D_18_/D_40_) embedded ribonucleotides were not cleaved by RNase HI, when incubated for 1 hr with *E. coli* RNase HI (NEB). Complete hydrolysis of the ribonucleotide containing strand of the substrate in A, C and D was achieved by heating in the presence of NaOH. (E and F) Hydroxyurea treatment to deplete cellular dNTPs, increases ribonucleotide incorporation by replicative polymerases in RnaseH2^null^ cells. Nucleic acids from untreated and HU-treated MEF cell lines. ‘+/+, +/−, −/− and −/−’ indicate *Rnaseh2b* genotypes of four independent MEF lines. RNase H2 treated total nucleic acids were denatured in formamide and separated by agarose gel electrophoresis. Co-treatment with RNase A was used to remove ribosomal RNA bands. Treatment of RNaseH2^null^ DNA (−/−) by RNase HI and/or formamide denaturation had no effect on fragmentation pattern (data not shown). (F) Densitometry of selected lanes from panel E. (G) RNaseH2^null^ cells are hypersensitive to low dose HU treatment with marked accumulation of cells in S phase. Propidium iodide FACS profiles. DNA content (*x* axis) plotted against cell number. Data shown: one representative experiment of 4 performed. (H) While using mitochondrial DNA as a control in our experiments (given its known alkali sensitivity), we made the observation that wild-type mitochondrial DNA, isolated from sucrose-gradient purified mouse liver mitochondria, can be fragmented by recombinant RNase H2 to a similar extent as by alkaline hydrolysis, but is not detectably cleaved by RNase HI. This is consistent with the accumulation of single (or double) ribonucleotides, indicating that for mitochondrial DNA ribonucleotide incorporation can be tolerated in a physiological context.

Treatment with recombinant RNase H2 enzyme also led to widespread fragmentation of RNaseH2^null^ genomic DNA, as shown by increased electrophoretic mobility after formamide denaturation. Significantly, the resulting fragmentation pattern was essentially identical to that seen after alkali treatment (Figure 6B), whereas no increased mobility was observed when inactive recombinant RNase H2 was used. In distinct contrast, recombinant *E. coli* type-1 RNase H (RNase HI) had no visible impact on the mobility of RNaseH2^null^ genomic DNA (Figure 6C). RNase HI efficiently digests substrates with four or more ribonucleotides but can also hydrolyze double-stranded nucleic acids with three embedded ribonucleotides, albeit at substantially lower rates (Hogrefe et al., 1990). Although the biological significance of this activity is unclear, oligonucleotide substrates with three ribonucleotides were cleaved efficiently under our assay conditions (Figures S5C and S5D). This activity was fully preserved in the presence of RNaseH2^null^ genomic DNA, ruling out any inhibitory effects that may be present within the nucleic acid preparation (Figures S5A–S5C). Thus, the differential sensitivity of genomic DNA from RNaseH2^null^ cells to RNase HI and RNase H2 activities established that the alkali and RNase H2 cleavable sites consist of one or, at most, two consecutive covalently incorporated ribonucleotides.

Quantitative analysis of the alkali-induced fragmentation permitted us to estimate the frequency of embedded ribonucleotides. Determination of DNA fragment distributions (Figure 5E) from the densitometry data (Figure 5D) predicted a rate of ribonucleotide incorporation of ∼1 in 7,600 nucleotides (nt) (analytical method described in Figure S4F). Additionally, the fragmentation pattern of hydrolyzed RNaseH2^null^ DNA lies between that generated by two nicking endonucleases, one that cuts, on average, once every 11 kb (7-cutter Nt.*Bsp*QI) and the other once every 3.7 kb (6-cutter Nb.*Bts*I) in the mouse reference genome (Figures 5F and 5G), supporting the computational estimate. Early and late passage MEFs exhibited similar levels of alkali sensitivity (data not shown). The incorporation of 1 ribonucleotide every 7,600 nucleotides during each round of replication would maintain such a steady-state level, so this is a minimum estimate for in vivo ribonucleotide misincorporation by polymerases.

Taken together, these results demonstrate the widespread presence of incorporated ribonucleotides in genomic DNA of RNaseH2^null^ MEFs. Ribonucleotide misincorporation was also evident in RNaseH2^null^ embryos (Figures 5C and S4E), consistent with this molecular defect underlying the developmental phenotype. Finally, we employed a chemical genetic approach analogous to previous yeast genetic experiments, in which a mutated Pol ɛ with an enhanced propensity for ribonucleotide incorporation was used (Nick McElhinny et al., 2010b). Cells were treated with a low dose of hydroxyurea (HU) to reduce cellular dNTP:rNTP ratios through partial inhibition of ribonucleotide reductase activity. This would then increase ribonucleotide incorporation by DNA polymerases. RNaseH2^null^ cells were observed to be hypersensitive to such a low dose of HU, accumulating in S phase (Figure S5G). Most significantly, such treatment resulted in additional alkali and RNase H2-sensitive sites in genomic DNA from RNaseH2^null^ cells (Figures 6D–6F, S5E, and S5F), consistent with such sites resulting from ribonucleotide misincorporation by DNA polymerases.

### Chromosome Instability in RNaseH2^null^ Cells

**Figure 7:**
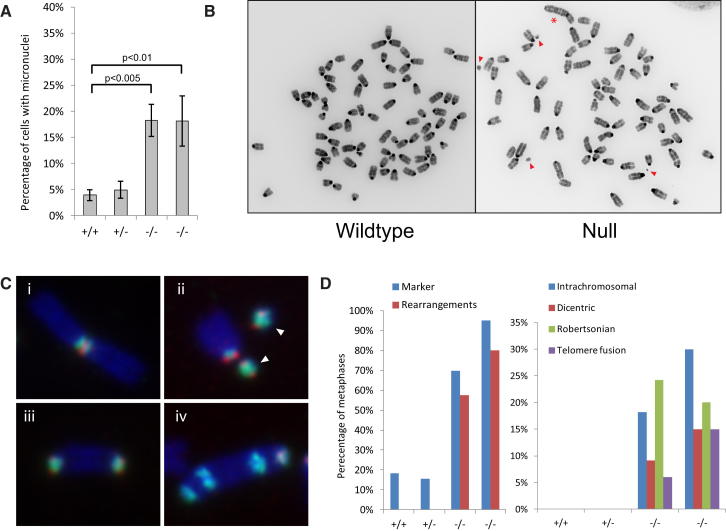
RNaseH2^null^ Cells Display Large-Scale Genome Instability (A) Micronuclei are frequently present in *Rnase2b^−/−^;p53^−/−^* cells. Error bars represent SD. n = 3 expts 500–1000 cells/expt. p value, t test. (B) Chromosomal rearrangements (asterisk) and marker chromosomes (arrowheads) are evident in DAPI-stained metaphase chromosomes of *Rnase2b^−/−^;p53^−/−^* MEFs. (C and D) FISH for major (green) and minor (red) satellite probes confirms the presence of frequent intrachromosomal translocations and heterochromatic minutes (marker chromosomes). (i) Robertsonian translocation, (ii) heterochromatic marker chromosomes (arrowheads), (iii) end-to-end translocation, and (iv) complex chromosomal rearrangement. (D) Quantification of cytogenetic anomalies identified in (C). n = 38, 32, 33, and 20 metaphases, respectively. p < 0.05 (Fisher's exact test) for all wild-type (+/+) versus mutant (−/−) comparisons. See also Figure S6.

**Figure S6:**
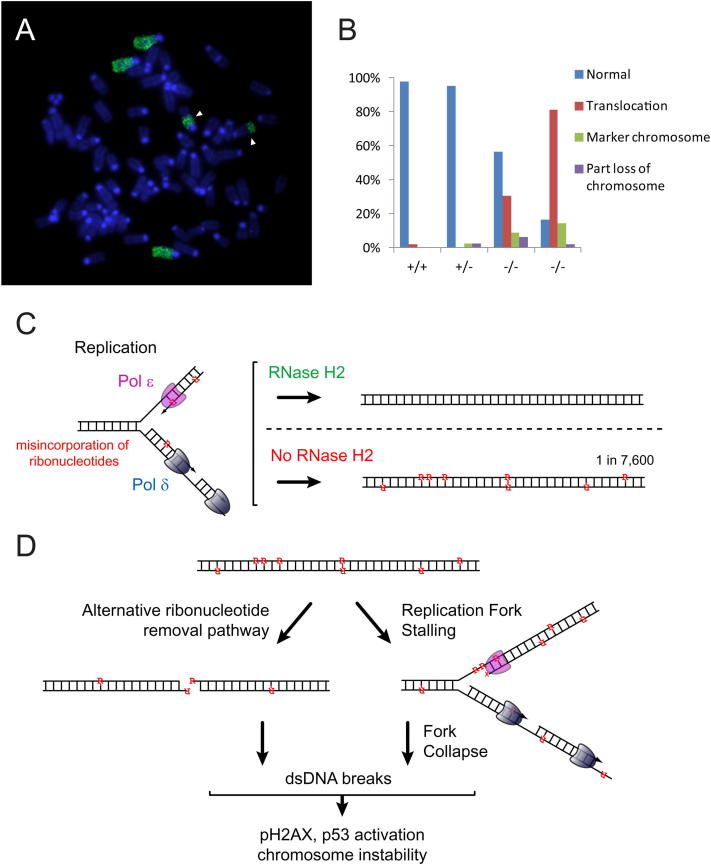
Chromosomal Abnormalities in RNaseH2^null^ Cells; A Model for Ribonucleotide Accumulation in RNaseH2^null^ Genomic DNA and Double-Strand Break Formation, Related to Figure 7 (A) Chromosome painting confirms the presence of cytogenetic rearrangements in RNaseH2^null^ MEFs. Image of metaphase with chromosome 4 paint showing a reciprocal translocation (arrow heads) and 3 intact copies of chromosome 4. (B) Quantification of abnormalities in chromosome 4, from metaphase chromosome painting. n = 46, 43, 46 and 48 metaphases respectively. (C and D) Model: Ribonucleotide accumulation in genomic DNA and double strand break formation (C) Ribonucleotides (R) are incorporated into genomic DNA by replicative polymerases (Pol δ, ɛ). RNase H2 initiates the removal of such nucleotides, a process that is likely to also involve FEN1 to generate a gap that is then repaired by a DNA polymerase and ligase. (D) In the absence of RNase H2 ribonucleotides accumulate on both DNA strands. Such ribonucleotides may cause replication fork stalling when clustered or when present in difficult to replicate genomic regions, leading to DNA damage response activation and proliferation arrest. Replication fork collapse could then lead to double strand break formation. Alternatively hydrolysis of directly opposed ribonucleotides may lead to double strand breaks, perhaps when processed by alternative mechanisms. Notably Topoisomerase I has appropriate site-specific ribonuclease activity to participate in such an alternative repair pathway (Kim et al., 2011; Sekiguchi and Shuman, 1997). ∼50 sites per RNaseH2^null^ cell where ribonucleotides are directly opposed would be expected, assuming a random distribution of ribonucleotides in genomic DNA.

Substantial levels of micronuclei, indicative of chromosomal breakage (Norppa and Falck, 2003), were observed in RNaseH2^null^ MEFs (Figure 7A), suggesting that the excessive presence of ribonucleotides in DNA causes large-scale genome instability. Likewise, large-scale cytogenetic anomalies were evident in DAPI-stained metaphase chromosomes (Figure 7B). Using satellite FISH probes, chromosomal rearrangements were present in virtually all metaphases from *Rnaseh2b^−/−^;p53^−/−^* MEFs, whereas they were only occasionally seen in control MEFs. Both minutes (marker chromosomes) and interchromosomal translocations were frequently observed, with translocations confirmed by FISH and chromosomal painting (Figures 7, S6A, and S6B).

## Discussion

### Ribonucleotides Accumulate in RNaseH2^null^ Cells as a Consequence of Incorporation by DNA Polymerases

Here, we report that substantial genome-wide incorporation of ribonucleotides occurs in mammalian genomic DNA and establish that RNase H2 is required for efficient removal of such nucleotides. Recent in vitro biochemical studies with yeast replicative polymerases have shown the misincorporation of ribonucleotides into DNA (Nick McElhinny et al., 2010a). Based on these findings, it was predicted that ribonucleotides may be incorporated into genomic DNA in vivo. Subsequent studies in both fission and budding yeast have established that this is indeed the case using alkali sensitivity assays of yeast genomic DNA (Miyabe et al., 2011; Nick McElhinny et al., 2010b). Here, we show that ribonucleotide incorporation also occurs in metazoans; demonstrate that such ribonucleotide lesions are harmful to mammalian cells; and establish that their removal is required for mouse embryonic development. Previous studies used an elegant genetic approach in which mutator replicative polymerases with increased propensity for ribonucleotide incorporation were used to infer the presence of incorporated ribonucleotides from enhanced alkali sensitivity of RNase H2 null yeast genomic DNA. Our findings provide further characterization of these alkali-sensitive sites by using enzymatic assays to directly substantiate that such lesions are single or diribonucleotides that are covalently incorporated into genomic DNA. Furthermore, we find that such lesions occur at a frequency of least 1,000,000 sites per cell, establishing them as the most common endogenous base lesions in the mammalian genome.

The presence of such ribonucleotides is most readily explained by misincorporation by the major replicative polymerases, which are estimated to incorporate one ribonucleotide every few thousand nucleotides in vitro (Nick McElhinny et al., 2010a). Alternatively, embedded ribonucleotides could result from failure to remove RNA primers during Okazaki-lagging strand processing. Such primers are ∼10 nt in length, much longer than the single/diribonucleotides found. However, retention of single ribonucleotides during this process remains a possibility (Rumbaugh et al., 1997). Theoretically, oxidation of deoxynucleotides that are present in DNA could also result in embedded ribonucleotides (Vengrova and Dalgaard, 2004), although this seems an unlikely explanation for their frequent occurrence.

Genomic DNA from RNase H2 null *S. cerevisiae* exhibits differential alkali sensitivity that correlates with the propensity of a mutant Pol ɛ to incorporate ribonucleotides (Nick McElhinny et al., 2010b). Here, we performed an analogous experiment using hydroxyurea to alter dNTP:rNTP ratios, favoring ribonucleotide incorporation. This promoted increased alkali and RNase H2 sensitivity, leading us to conclude that ribonucleotides that are embedded in genomic DNA are most likely the consequence of misincorporation by DNA polymerases. Given their frequent occurrence in genomic DNA, the predominant sources of such ribonucleotides are likely to be Pol ɛ and Pol δ, the major replicative polymerases that are responsible for leading- and lagging-strand synthesis, respectively (Burgers, 2009; Kunkel and Burgers, 2008; Miyabe et al., 2011). However, other DNA polymerases, such as Pol β (Cavanaugh et al., 2010) and lesion bypass polymerases, may also contribute.

### RNase H2 Is a Genome Surveillance Enzyme Required for Ribonucleotide Removal

Ribonucleotide accumulation in genomic DNA of RNaseH2^null^ mice (Figures 5 and 6) implicates the RNase H2 complex in the maintenance of genome integrity. This DNA repair function was originally suggested by Eder and colleagues (Eder et al., 1993). Ribonucleotides are likely to be harmful, as their ribose 2′-hydroxyl group increases susceptibility of the adjacent phosphodiester bond to hydrolysis. Specific patterns of mutations at nucleotide level have been observed in genomic DNA from RNase H2 null *S. cerevisiae* (Kim et al., 2011; Nick McElhinny et al., 2010a). Most frequently, these consist of 2–5 bp deletions, which are the result of topoisomerase-I-induced nicks at embedded ribonucleotides (Kim et al., 2011). However, the loss of yeast RNase H2 alone has a relatively small effect on mutation frequency, and the effect of embedded ribonucleotides on large-scale genome stability in yeast has not been reported. Therefore, the frequent occurrence of large-scale genome rearrangements in RNaseH2^null^ MEFs is unanticipated and noteworthy (Figure 7).

We estimate that ribonucleotides are incorporated at a rate of at least 1 every 7,600 nt in RNaseH2^null^ cells, corresponding to ∼1,300,000 lesions per cell. This is within the same order of magnitude predicted from in vitro incorporation rates by eukaryotic replicative polymerases (Nick McElhinny et al., 2010a) and is substantially higher than any other endogenous base lesions occurring in the mammalian genome. Even the previously most common lesions, such as abasic sites, 8-hydroxyguanine (8-oxoG), and 7-methylguanine, only occur up to 10,000 times per genome (Ciccia and Elledge, 2010; Lindahl and Barnes, 2000). As misincorporated ribonucleotides occur at at least 50-fold higher rates, without an efficient repair mechanism, they would be the most common noncanonical nucleotides present in mammalian DNA. Therefore, defining the processes that remove these ribonucleotides is of substantial interest. Of note, FEN1/Rad27 in conjunction with RNase H2 can excise ribonucleotides on an in vitro substrate, generating a single nucleotide gap on which a DNA polymerase and DNA ligase could act directly to repair the lesion (Rydberg and Game, 2002).

### Misincorporated Ribonucleotides Induce DNA Damage

In itself, ribonucleotide incorporation does not prevent replication: cellular proliferation is seen in both RNase H2 null mouse cells (*p53^−/−^*; Figure 4) and RNase H2 null budding yeast, in which p53 signaling is not evolutionarily conserved (Arudchandran et al., 2000; Belyi et al., 2010; Nick McElhinny et al., 2010b). DNA polymerases can tolerate templates containing ribonucleotides (Watt et al., 2011), which may explain why early embryogenesis in RNaseH2^null^ embryos proceeds normally. The absence of grossly perturbed transcriptional profiles later in development (Figure 4) suggests that mammalian RNA polymerases also tolerate ribonucleotide-containing templates.

However, excessive numbers of ribonucleotides do appear to be problematic. Replication fork stalling may occur in regions that contain clustered ribonucleotides, as seen at the *S. pombe* mating switch locus (Vengrova and Dalgaard, 2006). Incorporation of ribonucleotides in difficult to replicate regions or in close proximity to other lesions may be similarly detrimental. This is likely to explain the activation of DNA-damage response signaling observed in RNaseH2^null^ MEFs and embryos (Figures 4 and 5). Chromosomal rearrangements and micronuclei indicate the occurrence of double-strand DNA breaks. Such breaks may result from subsequent replication fork collapse or may be caused directly by hydrolysis of ribonucleotides on opposing DNA strands (see model in Figures S6C and S6D). Alternatively, the observed increase in PAR (Figure S4C) could suggest the presence of frequent single-strand breaks that would be converted at low frequency to double-strand lesions during replication. The accumulation of ribonucleotides in conjunction with rapid cell cycles in the epiblast (Snow, 1977) probably underlies the marked activation of DNA-damage signaling in the embryo. This then results in a p53-mediated inhibition of proliferation that is likely to substantially contribute to the lethality observed at E11.5 in RNaseH2^null^ embryos.

### Ribonucleotide Incorporation in Health and Disease

To our knowledge, stable incorporation of ribonucleotides has only been reported to date in two contexts. First, a diribonucleotide at the *S. pombe* mating switch locus is believed to be the signal initiating homologous recombination (Vengrova and Dalgaard, 2004). Second, the presence of ribonucleotides in mature mitochondrial DNA has been previously established (Grossman et al., 1973), and we now show these to be mono or diribonucleotides (Figure S5H). These sporadic ribonucleotides appear to be randomly distributed and thus are likely to result from replicative polymerase incorporation. The selectivity of the mitochondrial polymerase γ would be consistent with the presence of 10–30 ribonucleotides in mature mtDNA (Kasiviswanathan and Copeland, 2011). These may be tolerated by the mitochondrial genome either because of its relatively slow replication rate (Bogenhagen and Clayton, 1977) or owing to different mechanism(s) of genome replication (Clayton, 2003; Holt, 2009). Likewise, ribonucleotide incorporation is well tolerated in RNase H2-deficient *S. cerevisiae*, with normal viability and efficient cellular proliferation in unperturbed cells (Arudchandran et al., 2000; Nick McElhinny et al., 2010b). As recently reported, template switch and translesion DNA synthesis postreplication repair pathways may permit such ribonucleotides to be tolerated (Lazzaro et al., 2012). In contrast, in mice, we find that ribonucleotide removal is essential early in development. Similarly, mutation of other genes ensuring genome integrity, such as the catalytic subunit of pol ζ (Rev3), are viable in yeast but cause embryonic lethality at a similar stage in mutant mice (Esposito et al., 2000; Wittschieben et al., 2000). Such lethality may therefore be explained by the much larger size and complexity of the mammalian nuclear genome.

Low levels of ribonucleotide incorporation in the nuclear genome may be tolerated, and this could well be relevant to the autoinflammatory disorder Aicardi-Goutières syndrome (AGS), in which aberrant nucleic acid substrates are thought to drive an innate immune response (Crow and Rehwinkel, 2009). Reduced RNase H2 activity in AGS may therefore result in a chronic low level of ribonucleotide incorporation that is then processed by alternative (non-RNase H2 dependent) repair pathways. The increased levels of polyADPribosylation (Figure S4C), as well as the enhanced sensitivity to hydroxyurea observed in *Rnaseh2b^+/−^* MEFs (Figure S5G), could be consistent with this possibility. Aberrant nucleic acid species generated by such repair could then trigger an innate immune response. Alternatively, embedded ribonucleotides might induce DNA-damage response signaling that itself stimulates interferon production (Brzostek-Racine et al., 2011).

In summary, ribonucleotides are highly deleterious to the mammalian cell when left incorporated in the nuclear genome, causing substantial genome instability. RNase H2 is therefore a critical enzyme for ensuring the integrity of genomic DNA. Defining the pathway(s) that remove these ribonucleotides from genomic DNA, the site and nature of ribonucleotide-induced DNA damage, as well as the genome distribution of ribonucleotides will now be of substantial interest. This will help improve our understanding of the pathological and physiological roles of ribonucleotides in genomic DNA, of significance to both nucleic acid-driven autoimmunity and carcinogenesis.

## Experimental Procedures

### Generation of *Rnaseh2b* Null Mice

While performing targeted homologous recombination to generate 129/Ola ES cells with a c.520G > A (A174T) mutation in exon 7 of *Rnaseh2b* (that corresponds to the most common, hypomorphic AGS patient mutation, c.529G > A), we identified the *Rnaseh2b^E202X^* ES clone that had fortuitously acquired an additional mutation resulting in a premature stop codon: c.604G > T, E202X. Further details of gene targeting are available in the Supplemental Information. After karyotyping and blastocyst injection, resulting male chimeras were crossed to C57BL/6J females, giving rise to heterozygous *Rnaseh2b* knockin mice carrying both A174T and E202X mutations (*Rnaseh2b^tm1-hgu-A174T,E202X^*, elsewhere referred to as *Rnaseh2b^E202X^)*. Crosses with *p53^+/−^* mice (Clarke et al., 1993) were used to generate *Rnaseh2b^E202X/+^;p53^+/−^* double mutants. Knockout-first *Rnaseh2b* mice were generated by blastocyst injection of the *Rnaseh2b*^tm1a(EUCOMM)Wtsi^ ES cell clone EPD0087_4_A02 (EUCOMM ID: 24441; elsewhere referred to as *Rnaseh2b*^tm1a^) (Friedel et al., 2007). The *Rnaseh2b^tm1a^* allele is designed to prematurely truncate *Rnaseh2b* transcripts through targeted insertion into intron 4 of a genetrap cassette that contains a strong splice acceptor and an efficient polyadenylation signal (Testa et al., 2004).

### Genotyping

Genotypes of mice and embryos were determined by multiplex PCR. The status of early embryos was determined by α-RNase H2 immunofluorescence. For further details and primer sequences, see Supplemental Information and Table S1.

### RNase H Activity Assays

Enzyme activity assays were performed in triplicate using a FRET-based fluorescent substrate release assay as previously described (Crow et al., 2006a; Reijns et al., 2011) using 100 ng/μl of total protein from whole-cell extracts. RNase H2-specific activity was determined by subtracting the cellular activity against a sequence-matched DNA duplex without ribonucleotides.

### Western Blotting, Immunohistochemistry, Immunocytochemistry, and Microscopy

Immunoblotting was performed on whole-cell extracts as described previously (Crow et al., 2006a). For immunohistochemistry, tissues and deciduas were dissected into ice-cold PBS and fixed with 4% paraformaldehyde/PBS for 3–16 hr at 4°C with further processing performed by standard methods. Images were collected on Zeiss Axioplan II fluorescence or Nikon A1R confocal microscopes. For full experimental details, see the Supplemental Information; for antibodies and dilutions, see Table S2.

### Detection of Ribonucleotides in Genomic DNA

Total nucleic acids were isolated by mechanical disruption of MEFs or yolk sacks in ice-cold lysis buffer (20 mM Tris-HCl [pH 7.5], 75 mM NaCl, and 50 mM EDTA) and subsequent incubation with 100 μg/ml proteinase K, with Sarcosine then added to final 1% concentration. Nucleic acids were sequentially extracted with TE-equilibrated phenol, phenol:chloroform:isoamylalcohol (25:24:1), and chloroform; precipitated with isopropanol; washed with 75% ethanol; and dissolved in water. Mitochondrial DNA (mtDNA) was isolated from sucrose-gradient purified mitochondria as previously described (Pohjoismäki et al., 2010). For alkaline gel electrophoresis, total nucleic acids were heated for 2 hr at 55°C with 0.3 M NaOH and separated on agarose gels (50 mM NaOH, 1 mM EDTA) as previously described (Nick McElhinny et al., 2010b). Control samples were heated with 0.3 M NaCl and separated on 0.5× TBE agarose gels. Alternatively, nucleic acids were treated with RNase H enzymes and heated for 30–60 min in 90% formamide/20 mM EDTA at 37°C before separation on 0.5× TBE agarose gels. Digestions with RNase HI were carried out in 100 μl of 1× reaction buffer (NEB) with 5 U of enzyme for 1 hr at 37°C. Digestions with RNase H2 were carried out for 1 hr at 37°C in 100 μl reaction buffer (50 mM Tris [pH 8], 60 mM KCl, 10 mM MgCl_2_, 0.01% BSA, 0.01% Triton) using 10 nM of purified recombinant human RNase H2 (Reijns et al., 2011). Nucleic acids were ethanol precipitated and dissolved in 90% formamide/20 mM EDTA. After electrophoresis, gels were stained with SYBR Gold (Invitrogen) or ethidium bromide.

Extended Experimental Procedures **Generation of Rnaseh2b Null ES Cells**

A genomic fragment of 4.5 kb, which included exon 6 and 7 of the mouse *Rnaseh2b* locus (nucleotides 62977197-62981727 of Chr 14, Ensembl release 64), was retrieved by gap repair from BAC bMQ454F14 (Source Bioscience Lifesciences), and a floxed neomycin cassette was inserted between exon 6 and 7 by bacterial recombination as previously described (Martínez-Estrada et al., 2010), producing a targeting vector with two external homology arms of 2.8 kb and 1.7 kb. A point mutation c.520G > A (A174T) was introduced into Exon 7 of mouse *Rnaseh2b* by site-directed-mutagenesis (Quikchange II XL site directed mutagenesis kit, Agilent Technologies). The insert was removed from the vector backbone by digestion with *Not*I and SalI, purified by electroelution using an Elutrap (Schleicher & Schuell), and electroporated into 129/Ola E14Tg2AIV embryonic stem cells. Correctly targeted clones were selected by Southern blotting and long-range PCR. Exons 6 and 7 were sequenced and in one clone an additional c.613G > T mutation identified, which resulted in a premature stop codon (E202X). This ES cell clone was then karyotyped prior to injection into mouse blastocysts. All mouse studies were conducted under guidance and approval issued by the UK Medical Research Council and the UK Home Office.

**Genotyping**

For mouse genotyping, ear-clips were boiled in 50 μl 25 mM NaOH, 0.2 mM EDTA for 30 min at 95°C, cooled, then neutralized with 50 μl 40 mM Tris base and used directly in PCR reactions. For genotyping of embryos whole embryos, tails or yolk sacs were treated with DirectPCR Lysis Reagent (Viagen) according to the manufacturer's instructions. All genotyping PCRs were performed using a ‘multiplex’ three primer strategy and *Taq* ReddyMix PCR Master Mix (Thermo Scientific). For primer sequences, PCR programs and expected product sizes see Table S1. To genotype early embryos used for immunofluorescence, cryosections were stained with polyclonal affinity purified rabbit α-RNase H2 antiserum (raised against recombinant mouse RNase H2 complex; see Table S2). Embryos negative for RNase H2 staining were designated RNaseH2^null^; those positive for staining were used as RNase H2 positive controls.

**Generation of MEFs**

MEFs were isolated from individual E10.5 or E11.5 embryos after removing the head, by mincing in 1 ml of medium (DMEM, 10% FCS, 50 U/ml penicillin and 50 μg/ml streptomycin, 1 mM β-Mercaptoethanol). Resulting suspensions were grown at 37°C, 5% CO_2_ and 3% O_2_, and non-adherent cells removed after 24 hr.

**RNase H Activity Assays**

Enzyme activity assays were performed using a FRET-based fluorescent substrate release assay (Reijns et al., 2011). 10 μM of 3′ fluorescein-labeled oligonucleotides (GATCTGAGCCTGGGaGCT for RNase H2 specific activity, or gatctgagcctgggagct for total RNase H activity; uppercase DNA, lowercase RNA) were annealed to a complementary 5′ DABCYL-labeled DNA oligonucleotide (Eurogentec) in 60 mM KCl, 50 mM Tris-HCl pH 8, by heating for 5 min at 95°C followed by slow cooling to room temperature. Reactions were performed in 100 μl of buffer (60 mM KCl, 50 mM Tris-HCl pH 8, 10 mM MgCl_2_, 0.01% BSA, 0.01% Triton X-100) with 250 nM substrate in 96-well flat-bottomed plates at 24 ± 2°C. Fluorescence was read for 100 ms using a VICTOR2 1420 multilabel counter (Perkin Elmer), with a 480 nm excitation filter and a 535 nm emission filter.

**Cell-Cycle Analysis of RNase H2 Protein Levels**

HeLa cells were synchronized at the G1/S border by double thymidine block: 30%–40% confluent HeLa cells were incubated for 19 hr with 2 mM of thymidine, washed three times with PBS, incubated in fresh media for 9 hr, and incubated a second time with thymidine for 15 hr at 37°C, 5% CO_2,_ 21% O_2_. Cells were released from G1/S arrest by removal of thymidine, and harvested at 2 hr intervals for immunoblotting analysis. The cell cycle phase at each time point was determined by FACS analysis of propidium iodide stained cells fixed in 70% ethanol.

**Immunohistochemistry, Immunocytochemistry, and Microscopy**

For section immunohistochemistry, tissues and decidua were dissected into ice-cold PBS and fixed with 4% paraformaldehyde (PFA)/PBS for 3-16 hr at 4°C. After three 30 min washes with PBS, the samples were passed through a sucrose series, embedded, then frozen in OCT embedding medium (Sakura) and cryosectioned at 10 μM. Frozen sections were post-fixed in ice cold acetone 10 min at −20°C and air-dried prior to use; then washed three times in TBST (1x TBS, 0.1% Triton X-100). Sections were blocked with 10% donkey serum/TBST/1mM NaN_3_ for 1 hr at room temperature. Sections were incubated with diluted primary antibody (Table S2) in 1% donkey serum/TBST/1mM NaN_3_ for 14-16 hr at 4°C. Slides were washed three times 10 min with TBST before incubating with secondary antibody (Table S2) diluted in 1% donkey serum/TBST/1mM NaN_3_ for 4 hr at room temperature with TOTO-3 (Invitrogen, 1:400) and RNase A (50 μg/ml). Samples were washed three times 10 min in TBST and mounted in Prolong Gold (Molecular Probes).

For cell proliferation studies, pregnant dams and postnatal animals were given a single intraperitoneal injection of EdU (Invitrogen) at a dose of 50-100 mg/kg body weight in a solution of 10 mg/ml PBS (pH 7.4). After 1-2 hr, animals were sacrificed and tissues harvested and fixed in 4% PFA/PBS with gentle mixing for 3 hr at 4°C and processed as described above. Click-iT reactions (Invitrogen) were performed on cryosections as per manufacturer's instructions.

For whole-mount immunohistochemistry of blastocysts, E2.5 embryos were collected from *Rnaseh2b^+/−^* x *Rnaseh2b^+/−^* plugs and cultured overnight in M16 media (Sigma) to blastocyst stage. Blastocysts were then fixed in 4% PFA/PBS for 20 min and transferred to depression slides for subsequent steps. Blastocysts were washed five times 5 min in PBST (1x PBS, 0.1% Triton X-100), permeabilized in PBS/0.25% Triton X-100 for 20 min and washed five times 5 min in PBST. Blastocysts were blocked in 10% donkey serum/TBST/1 mM NaN_3_ for 2 hr at room temperature and incubated 14-16 hr in primary antibody (Table S2) diluted in 1% donkey serum/TBST/1 mM NaN_3_ in a humidified chamber at 4°C. Samples were washed three times 10 min in TBST, prior to incubating with secondary antibody (Table S2) diluted in 1% donkey serum/TBST/1 mM NaN_3_ for 4 hr at room temperature with TOTO-3 (Invitrogen, 1:400) and RNase A (50 μg/ml). Samples were washed three times 10 min in TBST and mounted on a Superfrost slide between 9 mm Secure-Seal spacers (Molecular Probes) and no. 0 coverslips in Prolong Gold (Molecular Probes). Details of antibodies and dilutions used in this study are given in Table S2.

Images were collected on a Zeiss Axioplan II fluorescence microscope or on a Nikon A1R confocal microscope, comprised of a Nikon Eclipse TiE motorized inverted microscope with Perfect Focus System. Image capture and analysis were performed using IPLab Spectrum (Scanalytics Corp) driving a Coolsnap HQ CCD camera (Photometrics Ltd) for the Zeiss system. For the confocal, image capture and analysis was performed using Nikon NIS Elements AR.

**Illumina Microarray Transcriptome Analysis**

RNA was extracted from individual embryos using TRIzol reagent (Invitrogen), with further purification using RNeasy Mini Kit columns (QIAGEN) and RNase-free DNase I (QIAGEN). cRNA was generated using an Illumina TotalPrep RNA Amplification Kit (Ambion) and whole-genome gene expression analysis of biological triplicates performed using MouseWG-6 v2.0 Expression BeadChips (Illumina). MIAME compliant microarray data was deposited at the Gene Expression Omnibus (GEO, http://www.ncbi.nlm.nih.gov/geo/) under the accession number GSE37419.

**Microarray Statistical Analysis**

Microarray data were analyzed with R 2.14.0, using its beadarray (Dunning et al., 2007) and Limma 3.10.2 (Smyth, 2004) packages. Raw, non-normalized bead-summary values were imported from the Illumina BeadStudio software into R using the beadarray package. Quantile normalization was applied to the data to enable comparison between arrays. A linear model was applied to the expression data for each gene. To determine statistically differentially expressed genes the results of the linear model were summarized and a Bayes moderated t test applied. To control for multiple testing, a Benjamini and Hochberg false discovery rate p-value of < 0.05 was used.

**qPCR**

10 μl reactions consisting of 1 μl template cDNA, 1 X Brilliant II Sybr Green qPCR Master Mix (Stratagene), 0.3 μM passive reference dye (ROX, Stratagene) and either 0.1 μM of each oligonucleotide primer (Ccng1, Cdkn1a) or 0.06 μM of each primer (Actb) in nuclease-free water. Each sample and -RT were analyzed in triplicate and a no template control was include for each master mix. Quantitative real-time PCR was performed using an ABI Prism HT7900 Sequence Detection System (Applied Biosciences) with the following protocol: 50°C (2 min), 95°C (10 min) followed by 40 cycles of 95°C (15 s), 60°C (1 min). The cycle threshold (CT) values for each gene were used to calculate the expression of each target gene normalized to beta-Actin using the comparative CT method (Livak and Schmittgen, 2001). Specificity of the primers was confirmed by agarose gel electrophoresis of the PCR products.

**Mathematical Modeling of Ribonucleotide Incorporation Rate**

Numeric and simulation analysis was performed in R (version 2.10.1). Peaks in the size reference lane were identified under supervision and the linear model lm(y∼log(x)) fitted to produce an electrophoretic distance (y) to nucleotide size (x) calibration curve. Densitometric histograms of electrophoresis lanes (e.g., Figure 5D) corresponding to alkali-treated genomic DNA were smoothed by fitting the smooth.spline function with 40 degrees of freedom. These smoothed distributions were transformed into nucleotide coordinate based histograms using a calibration curve from the same gel derived from 1 kb size standards (Invitrogen), extrapolated where necessary. Histograms were scaled such that they represented genomic fragment size distributions summing to 10^9^ nucleotides. Direct subtraction of the number of genomic fragments in each histogram provides an approximate analytic estimate of the difference in fragment number between lanes. Simulation studies were based on the fragmentation of 10^9^ nucleotide genomes. Random cuts were added to a starting genome, either cut at random or with a starting distribution of cuts estimated from densitometric data as above. A hill climbing strategy with 50 iterations was employed to find the optimal number of random cuts added, to achieve the best fit with a target histogram (e.g., turning the *Rnaseh2b^+/+^* densitometric distribution into the *Rnaseh2b^−/−^* distribution). The objective function for optimization was to minimize the Manhattan distance between the target histogram and the histogram of the simulated data after smoothing as above.

**FACS Analysis**

Mouse embryonic fibroblasts (10^6^) were seeded into T175 flasks and incubated for 14-16 hr at 37°C, 5% CO_2_ and 3% O_2_. Cells were cultured for another 48 hr in the absence or presence of hydroxyurea (300 μM). Cells were subsequently trypsinized, washed in PBS and fixed in 70% ice cold ethanol. Fixed cells were pelleted and resuspended in 2 ml pre-warmed pepsin (Sigma) dissolved at 1 mg/ml in 30 mM HCl and incubated at 37°C for 30 min with frequent mixing. Cell nuclei were pelleted at 3350 g for 4 min and resuspended in 100 μl 0.1 mg/ml RNase A diluted in PBS-EDTA before the addition of 100 μl propidium iodide (100 μg/ml) diluted in PBS-EDTA. Each sample was incubated at 4°C for a minimum of 1 hr before acquisition on a BD Biosciences FACSAriaII. Data was analyzed using FlowJo software (v7.6.1, Tree Star).

**FISH of Metaphase Chromosomes**

For 2D FISH, metaphases were isolated in hypotonic buffer, fixed with methanol:acetic acid (3:1) and dropped onto slides. Slides were incubated with 100 μg/ml RNase A in 2x SSC for 1 hr at 37°C, washed in 2x SSC and dehydrated through an alcohol series, followed by denaturation in 70% formamide/2x SSC at 70°C. Major (pSAT) (Okano et al., 1999) and minor (R198) (Kipling et al., 1994) satellite probes were prepared and labeled by nick translation with digoxigenin-11-dUTP or biotin-16 dUTP. Probes (100 ng per slide) were mixed with 5 μg of sonicated salmon sperm DNA, denatured and hybridized to slides for 14-16 hr at 37°C. For chromosome 4 painting, 15 μl per slide of Ready-to-use use paint (Cambio) was denatured at 70°C for 5 min, re-annealed at 37°C for 15 min and hybridized to denatured slides for 14-16 hr at 37°C. Washes and detection were performed as previously described (Morey et al., 2007). Two-dimensional slides were examined using a Zeiss Axioplan II fluorescence microscope with Plan-neofluar objectives, a 100 W Hg source (Carl Zeiss) and Chroma #8300 triple band pass filter set (Chroma Technology Corp.) with the excitation filters installed in a motorized filter wheel (Ludl Electronic Products). Grayscale images were captured with a Hamamatsu Orca AG CCD camera (Hamamatsu Photonics Ltd.). Image capture and analysis were performed using in-house scripts written for IPLab Spectrum (Scanalytics Corp.).

**Statistical Analysis**

For embryonic and live-born viability, categorical data was analyzed using Chi-square tests, under the null hypothesis that mutant mice would be present at Mendelian ratios. All quantitative data was analyzed using Student's t tests, under the assumption that data followed a Normal distribution. For cell counting experiments (e.g., Figure 4C, 5B and 7A), the percentage of positive cells was calculated for each experiment. This continuous measure was then analyzed by unpaired t tests, to establish if differences between cell lines were statistically significant, allowing for inter-experimental variation.

## Accession Numbers

Microarray data for E9.5 *Rnaseh2b^E202X/E202X^* and wild-type embryos have been deposited in the Gene Expression Omnibus under the accession number GSE37419.

## Supplemental Information

Document S1. Tables S1 and S2

Document S2. Article plus Supplemental Information
