# Supplementary material for: Enzymatic Removal of Ribonucleotides from DNA Is Essential for Mammalian Genome Integrity and Development
Source: Cell. 2012 May 25;149(5):1008–22. doi: 10.1016/j.cell.2012.04.011 (PMC3383994; doi:10.1016/j.cell.2012.04.011)
Supplement: Document S1. Tables S1 and S2 [file mmc1.pdf]

Table S1. PCR Primers, Related to Experimental Procedures

Name	Sequence	Description
**Primer a**	GATAGCTGACAAAGATAACTC	*Rnase2b*^{E202X} multiplex PCR genotyping (WT product 221 bp, mutant product 420 bp; see Figure 1)[1]
**Primer b**	CTGTCCATCTGCACGAGACT	
**Primer c**	CCTGGAAACCTGACCACC	
**p53forw**	GTGGTGGTACCTTATGAGCC	*p53* multiplex PCR genotyping (WT product 642 bp, mutant product 510 bp)[2]
**p53rev1**	CAAAGAGCGTTGGGCATGTG	
**p53rev2**	CATCGCCTTCTATCGCCTTC	
**GTBforw**	GACCTGGGCAGACAGTGAAT	*Rnase2b*^{tm1a} multiplex PCR genotyping (WT product 448 bp, mutant product 519 bp)[1]
**GTBrev1**	CCACAACGGGTTCTTCTGTT	
**GTBrev2**	GAGCCAGCTGAGGACTCAAG	
**Probe-F**	TTAGAAGCAGCAAGTGAGC	PCR amplification of ~400 bp Southern probe (see Figure 1)
**Probe-R**	CAAGGACTTGCAGAATGTAG	
**ArmI-F**	GTCTAGCTCAATGATTCACAAG	For ES cell genotyping, detecting correct targeting of *Rnase2b*^{E202X} (see Figure 1)
**ArmI-R**	CCCGGTAGAATTTCGACG	
**ArmII-F**	ATCGCATTGTCTGAGTAGGTG	ES cell genotyping, detecting correct targeting of *Rnase2b*^{E202X} (see Figure 1)
**ArmII-R**	TGCCAGGTGCCAATCACAG	
**Rnaseh2b-F**	AAGTACAGCTCAGAGAAGACATTGAA	Rnaseh2b qPCR
**Rnaseh2b-R**	CTTTTAGTGCCACCACAGTTTG	
**Actb-F**	CTAAGGCCAACCGTGAAAAG	β-Actin qPCR
**Actb-R**	ACCAGAGGCATACAGGGACA	
**Cdkn1a -F**	TCCACAGCGATATCCAGACA	Cdkn1a/p21 qPCR
**Cdkn1a -R**	GGACATCACCAGGATTGGAC	
**Ccng1-F**	TTAGTAGGCCTGTCGGATCG	Cng1/Cyclin G1 qPCR
**Ccng1-R**	AGCAGTTTCTGAGAGTCAGTTGTC	

[1]PCR program: 95°C 2 min; then 30 cycles of 94°C 45 s, 58°C 45 s and 72°C 45 s; then 72°C 5 min

[2]PCR program: 95°C 5min; then 3 cycles of 94°C 30 s, 65°C 30 s and 72°C 45 s; then 3 cycles of 94°C 30 s, 62°C 30 s and 72°C 45 s; then 3 cycles of 94°C 30 s, 59°C 30 s and 72°C 45 s; then 35 cycles of 94°C 30 s, 56°C 30 s and 72°C 45 s; then 72°C 10 min

Table S2. Antibody Reagents, Related to Experimental Procedures

Antigen	Antibody/ Clone Name	Host Species	Dilution	Applications	Source
**Actin**	A2066	Rabbit	1:2,000	WB[1]	Sigma
**β-catenin**	15B8	Mouse	1:500	IF[2], f-IHC[3]	Sigma
**Ki67**	TEC-3	Rat	1:50	IF, f-IHC	Dako
**p21**	F-5	Mouse	1:100	WB	Santa Cruz
**PAR**	4336-APC	Rabbit	1:2,000	WB	Trevigen
**Phospho-Histone H3 (Ser10)**	06-570	Rabbit	1:5,000	WB	Millipore
**Phospho-Histone H2A.X (Ser139)**	2577	Rabbit	1:50	IF, f-IHC	Cell Signaling Technology
**Phospho-Histone H2A.X (Ser139) (20E3) Alexa 488 Conjugate**	9719	Rabbit	1:50	IF, f-IHC	Cell Signaling Technology
**RNASEH2A**	TA306706	Rabbit	1:1,000	WB	Origene
**mouse RNase H2 complex[4]**	62	Rabbit	1:100-500	IF, f-IHC, WB	See Experimental Procedures
**human RNase H2 complex[4]**	7	Sheep	1:100-500	WB	See Experimental Procedures

Secondary antibody reagents				
**α-Mouse IgG, HRP-conjugated**	Goat	1:5,000	WB	Dako
**α-Rabbit IgG, HRP-conjugated**	Goat	1:5,000	WB	Cell Signaling Technology
**α-Sheep IgG, HRP-conjugated**	Rabbit	1:2,000	WB	Dako
**Alexa 488, 594 or 647-conjugated α-Mouse IgG**	Donkey	1:400	IF, f-IHC	Molecular Probes
**Alexa 488-conjugated α-Rat IgG**	Donkey	1:250	IF, f-IHC	Molecular Probes
**Alexa 488, 594 or 647-conjugated α-Rabbit IgG**	Donkey	1:400	IF, f-IHC	Molecular Probes

[1]WB = Western Blotting

[2]IF = Immuno-fluorescence

[3]f-IHC = Fluorescent Immunohistochemistry

[4]Polyclonal antibodies were raised against recombinant mouse and human RNase H2 complexes, purified as previously described (Reijns et al., 2011), and affinity purified.
